# Environmental disturbances and cyanobacterial traits shape prokaryotic dynamics in a eutrophic Mediterranean coastal lagoon

**DOI:** 10.1186/s40793-026-00893-9

**Published:** 2026-04-29

**Authors:** Ana M. Cabello, Soluna Salles, Guillermo Domínguez-Huerta, Eric Capo, M. Teresa Camarena-Gómez, Candela García-Gómez, Antonio Sánchez, Jean-François Mangot, Isabel Cerezo, Rocío Bautista, Patricia Pérez, Rocío García, Juan M. Ruiz, Jesús M. Mercado, Isabel Ferrera

**Affiliations:** 1 Centro Oceanográfico de Málaga, Instituto Español de Oceanografía (IEO-CSIC), 29002 Málaga, Spain; 2 Department of Ecology and Environmental Science, Umeå University, Umeå, Sweden; 3 Department of Marine Biology and Oceanography, Institut de Ciències del Mar (ICM-CSIC), 08003 Barcelona, Spain; 4 Andalusian Platform of Bioinformatics-SCBI, University of Málaga, Málaga, Spain; 5 Centro Oceanográfico de Murcia, Instituto Español de Oceanografía (IEO-CSIC), 30740 San Pedro del Pinatar, Murcia Spain; 6 Present Address: Ministerio para la Transición Ecológica y el Reto Demográfico, 28003 Madrid, Spain

**Keywords:** Mar Menor, Eutrophication, Coastal lagoon, Amplicon sequencing, Metagenome-assembled genomes, *Synechococcus*, Prokaryotic microbiome

## Abstract

**Background:**

Coastal ecosystems face increasing threats from eutrophication, driven by excess nutrient inputs that lead to ecosystem-disruptive algal blooms (EDABs). The Mar Menor coastal lagoon, located in the south-eastern Iberian Peninsula, has experienced severe ecological disruption since 2015, beginning with a *Synechococcus*‑dominated cyanobacterial bloom and followed by major shifts in eukaryotic phytoplankton composition. However, the mechanisms that affect phytoplankton dynamics in this coastal environment remain unknown. Here, we investigate the spatiotemporal dynamics of prokaryotic communities in the lagoon after the initial *Synechococcus* bloom using three years of 16S rRNA gene sequencing data and evaluate how environmental factors shape these patterns. In addition, we examine the fine‑scale diversity and dynamics of *Synechococcus* variants through metagenomics (*petB* gene) and use genome‑resolved analyses to identify functional traits associated with their succession in the lagoon. Finally, to investigate the role of biotic interactions in regulating cyanobacterial growth, we examine the temporal dynamics of cyanophages.

**Results:**

Microbial communities in the waters of the Mar Menor responded rapidly and consistently to short‑term environmental fluctuations and showed a weak seasonal signal in alpha and beta diversity. Prokaryotic assemblages associated with two deoxygenation events following extreme weather conditions (intense rainfall in autumn 2019 and unusually high temperatures in summer 2021) illustrated how episodic disturbances can drive substantial shifts in microbial composition; notably, *Synechococcus* became particularly prevalent after the intense rainfall event. Fine‑scale analyses of 16S rRNA and *petB* gene variants revealed that a restricted set of *Synechococcus* lineages dominated throughout the study period. Comparative genomic analyses of these cyanobacterial populations highlighted distinct functional repertoires, including genes involved in osmoprotectant biosynthesis, diverse toxin-antitoxin systems, herbicide resistance, and multiple viral defense mechanisms, present only in specific variants. Finally, temporal analyses of viral assemblages indicated that cyanophages played a key role in modulating *Synechococcus* population dynamics.

**Conclusions:**

The temporal dynamics of prokaryotic communities in the Mar Menor indicate that the lagoon remains in an altered, non‑equilibrium state, likely sustained by recurrent anthropogenic and climatic pressures. The contrasting microbial responses observed during two different deoxygenation events underscore the ecosystem’s complexity. This study highlights the importance of incorporating microbial community analyses into long‑term monitoring of threatened coastal systems, and the power of comparative genomics for identifying functional traits that enable cyanobacterial proliferation in disturbed ecosystems.

**Supplementary Information:**

The online version contains supplementary material available at 10.1186/s40793-026-00893-9.

## Background

Coastal ecosystems, among the planet's most productive and biodiverse environments, are increasingly threatened by pressures arising from both natural processes and human activities [1, 2]. Eutrophication of coastal areas is a growing environmental concern with severe ecological and economic consequences for affected systems [3]. Eutrophication is driven by an excess of nutrients that stimulates primary production, reduces water transparency, and increases the amount of organic matter (DOM) in the ecosystem. This process has been linked to the rising frequency of phytoplankton blooms, particularly ecosystem disruptive algal blooms (EDABs), which can profoundly alter ecosystem structure and function [4–7]. The large quantities of DOM released by these algal blooms are subsequently remineralized by heterotrophic bacteria [8–12]. The degradation of senescent blooms by these bacteria can lead to oxygen depletion and, under extreme conditions, anoxia [13]. Such oxygen‑deficient environments can drive substantial shifts in biodiversity, disrupt biogeochemical cycles, and alter ecosystem functioning long after the initial bloom event fades [14–17].

The species responsible for these algal blooms across different regions belong to diverse taxonomic groups but share common traits, including small cell size compared to other phytoplankton species and, potentially, the capacity to utilize organic forms of nutrients [5, 6]. Importantly, their ability to assimilate inorganic nutrients does not appear to exceed that of other bloom-forming taxa [5]. Despite these shared features, the specific environmental or ecological factors that trigger EDABs remain poorly understood. Over the last decade, advances in high-throughput DNA sequencing technologies have allowed the characterization of microbial communities with unprecedented resolution, allowing the identification of individual taxa [18, 19]. More recently, metagenomics and genome-resolved analyses further enable the identification of functional traits and metabolic capacities that may allow EDAB species to proliferate under particular environmental conditions, helping understand the mechanisms underlying these blooms [20–22].

A notable example of a coastal environment that has experienced severe ecological deterioration over the past decade is the Mar Menor, the largest hypersaline coastal lagoon in Europe, where one of the most relevant EDABs in the Mediterranean Sea was reported [23, 24]. Historically, the Mar Menor was considered an oligotrophic system, characterized by low chlorophyll *a* (Chl *a*) concentrations in its water column and salinity values ranging between 42 and 47 [1, 25, 26]. At the end of 2015, the lagoon experienced a drastic decrease in transparency, followed by a dramatic increase in Chl *a* concentration, leading to the phenomenon known as ‘green soup’ in 2016 [23]. Seawater sampling for flow cytometry analyses combined with satellite color images, indicated that the picocyanobacterium *Synechococcus* played a significant role in the early stages of this event’s development [7]. Signs of ecosystem deterioration (i.e. increased chlorophyll concentrations, reduced water transparency, and declining macrophyte communities) persisted in the lagoon for several months after the *Synechococcus* bloom, with indications of recovery emerging in the first half of 2018 [24]. However, new episodes of rapid phytoplankton proliferation followed by extensive deoxygenated zones occurred in October 2019 and August 2021 [27, 28]. Microscopy analyses confirmed that multiple species of diatoms contributed substantially to these later blooms [27]. Yet, it remains unclear whether *Synechococcus* also participated in these subsequent events or which factors govern the magnitude of its growth. Although fluctuations in environmental variables such as nutrient availability, temperature, and salinity are likely contributors, biotic interactions may also exert a significant influence [29–32]. Indeed, genomic comparisons have shown that functionally diverse populations and virus-host interactions shape the dynamics of cyanobacterial harmful algal blooms in lake ecosystems [33]. Whether similar mechanisms affect bloom dynamics in coastal environments such as the Mar Menor remains unknown. Nevertheless, the recurrent bloom episodes in the lagoon highlight the need to identify the environmental and biological drivers of such ecological perturbations, in order to understand bloom dynamics, and ultimately enhance our capacity to anticipate and mitigate future ecosystem disruptions.

This study aims, first, to characterize the spatiotemporal dynamics of prokaryotic communities in the Mar Menor coastal lagoon and evaluate how environmental variables structure these communities, and second, to identify the ecological processes and biological interactions that modulate picocyanobacterial bloom formation. To achieve these goals, we first analyze the composition of the prokaryotic community at amplicon sequence variant (ASV) resolution over a three-year period using 16S rRNA gene sequencing and assess whether the observed changes are influenced by environmental seasonality. Next, to explore the fine-scale dynamics of picocyanobacterial genetic variants, we use metagenomics and examine variation in the *petB* gene, a functional marker gene that provides higher phylogenetic resolution than the 16S rRNA gene for photosynthetic organisms. Once the diversity of the picocyanobacterial community is resolved, we reconstruct metagenome-assembled genomes (MAGs) of *Synechococcus* populations to gain insights into the functional traits that may explain their ability to thrive in this coastal lagoon. Finally, to investigate the role of biotic interactions in regulating cyanobacterial growth, we examine the temporal dynamics of their associated cyanophages. By integrating community profiling, genome‑resolved metagenomics, and phage dynamics, these analyses not only identify, for the first time, the temporal succession of prokaryotic communities in the waters of the Mar Menor, but also offer a comprehensive perspective on the factors that govern picocyanobacterial growth in this coastal lagoon.

## Material and methods

### Study site and sampling procedures

**Fig. 1:**
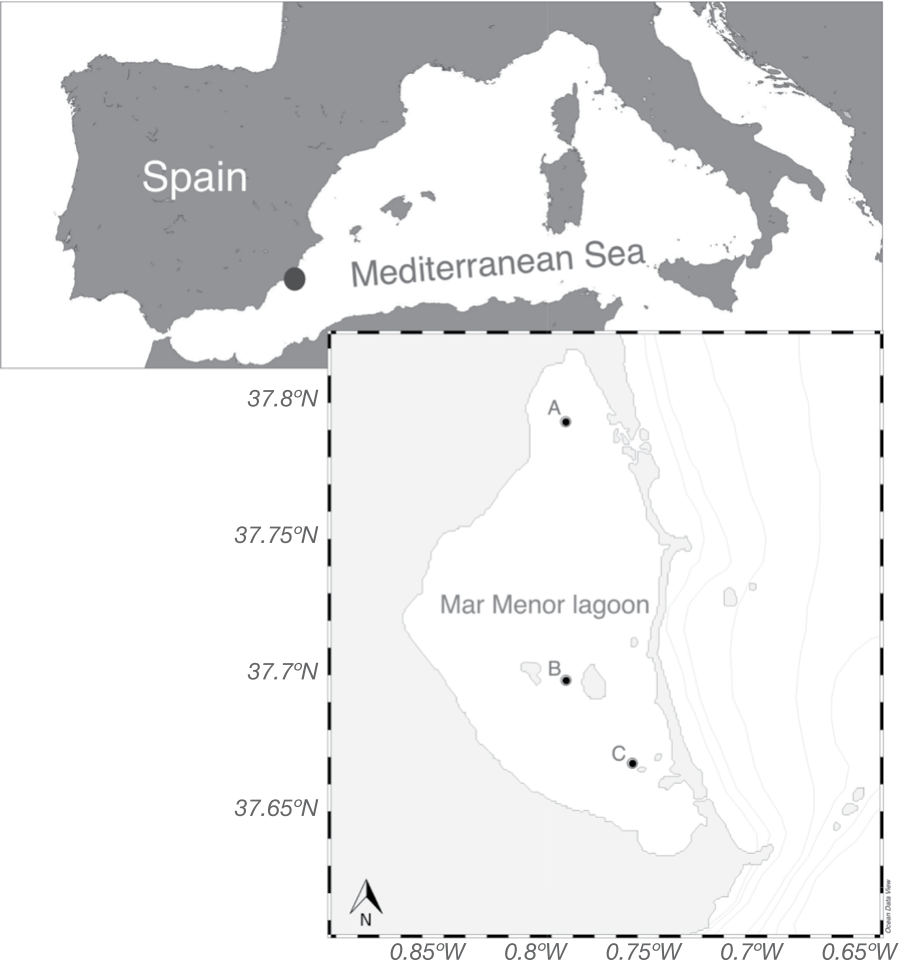
Map of the Mar Menor coastal lagoon showing the location of the three sampling stations (A, B, and C)

This study was conducted in the Mar Menor coastal lagoon, located in the southwestern Mediterranean Sea, off the coast of the Murcia province (southeastern Spain). Water samples and ancillary data were obtained as part of a time series monitoring program run since 2016 by the Spanish Institute of Oceanography at three locations in the lagoon, A, B, and C (Fig. 1).

Our study covers a three-year period, from October 2019 to October 2022 (note that sampling could not be performed during the COVID-19 lockdown, i.e., from March 15th to June 21st 2020). At each station, hydrological variables (temperature, salinity, oxygen, pH, and Photosynthetically Active Radiation [PAR] irradiance) were measured at surface and at an intermediate depth of 4 m (note that the bottom depth at the sampling stations is 6–7 m). Light extinction coefficients (K_d_) were estimated from PAR profiles. Water samples for analyses of chemical and biological variables were collected at 4 m depth, which is representative of the entire water column as previous observations have shown that the lagoon is generally not stratified [34]. Chlorophyll *a* (Chl *a*) concentrations, dissolved nutrient concentrations, including inorganic (nitrate, nitrite, ammonia, phosphate, and silicate) and organic forms (total N and total P), along with picoplankton cell counts were obtained monthly. Sampling procedures and environmental data acquisition for these variables are described in detail elsewhere [7, 27]. Cell abundances for photosynthetic picoeukaryotes (PPEs), cyanobacteria, and heterotrophic bacteria were estimated from triplicate samples by flow cytometry using a Becton Dickinson FACScan flow cytometer in samples fixed with 1.5% glutaraldehyde (final concentration). Cell counting for autotrophic picoplankton was performed based on the forward-light scatter (FLS) and the orange and red fluorescence signals. For heterotrophic bacteria, samples were stained with SYBR Green I (Sigma-Aldrich) (10× final concentration) and counted based on their relative green fluorescence (FL1) and side-scatter (SSC) signals. Absolute counts were calculated by estimating the flow rates using TruCount™ bead suspensions prepared by adding deionized water to TruCount™ tubes (Becton Dickinson).

### DNA sample collection, extraction, and sequencing

**Fig. 2:**
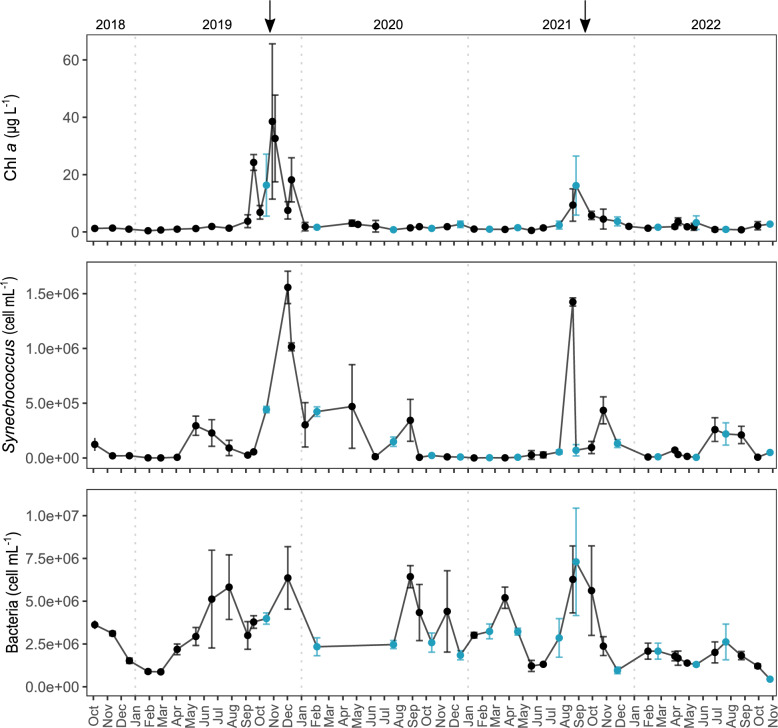
Monthly chlorophyll *a* concentrations and cell abundances of *Synechococcus* and heterotrophic bacteria measured by flow cytometry. Values represent averages from stations A, B, and C at 4 m depth, with error bars indicating standard deviations. Sampling months are shown on the x-axis, and the corresponding year is indicated above the top panel. Blue dots mark the dates on which samples for DNA analyses were collected. Arrows above the top panel indicate the two deoxygenation events that occurred in October 2019 and August 2021. Part of the chlorophyll *a* concentration and *Synechococcus* abundance data (up to 2021) were previously published [7, 27]

Seawater samples for DNA collection were taken seasonally, covering three seasonal cycles and resulting in a total of 42 samples from three stations across 14 sampling dates (see blue dots in Fig. 2). Samples were prefiltered by a 200 µm nylon mesh and sequentially filtered through 3 µm and 0.2 µm pore-size polycarbonate membrane filters (47 mm diameter, DHI) using a peristaltic pump, in order to target the microbial community within the picoplankton size range (i.e. 0.2–3 µm). In most cases, between 0.6 and 1 L of seawater could be filtered at each station before filter clogging occurred. Filters were preserved at − 80 °C until DNA extraction.

DNA was extracted from the 0.2 µm filters using the DNeasy PowerSoil Pro Kit® (QIAGEN). Filters were cut into pieces using sterile razor blades before being processed following the manufacturer’s instructions. The quality of the extracted DNA was evaluated by spectrophotometry using a BioDrop Touch Duo (µLite 0.5 mm cuvette; Biochrom). Accurate DNA quantification was performed through fluorometry utilizing the Qubit technology (Life Technologies). The extracted DNA was stored at − 80 °C until further analysis.

To study the prokaryotic community, we used a metabarcoding approach targeting the V4-V5 region of 16S rRNA gene, using primers 515F-Y (5'-GTGYCAGCMGCCGCGGTAA-3′) and 926R (5'-CCGYCAATTYMTTTRAGTTT-3′) [35]. The resulting amplicons were sequenced using an Illumina NovaSeq 6000 PE250 platform (AllGenetics; www.allgenetics.eu). This approach was applied to the 42 samples collected from the three stations. In addition, a shotgun metagenomic sequencing approach was performed on the DNA extracts from 14 samples from Station B (Fig. 1) using an Illumina NovaSeq 6000 PE150 platform (Novogene; www.novogene.com).

### Metabarcoding read processing

Primers and spurious sequences from the amplicon sequencing data were trimmed using cutadapt 4.4 [36]. Trimmed reads were subsequently processed using DADA2 v.1.22 [37] with default parameters to define amplicon sequence variants (ASVs) and remove chimeric sequences. Taxonomic assignment of the ASVs was performed within DADA2 with the Ribosomal Database Project Bayes (RDP) naïve classifier, implemented against the SILVA v138.1 database [38]. ASVs classified as mitochondria or chloroplasts were subsequently removed. The resulting ASV table was randomly subsampled to 69,632 reads per sample, retaining a total of 6,819 ASVs. Those ASVs assigned to *Synechococcus* were further checked against the Cyanorak database v2.1 [39] through 100% BLAST matches.

### Metagenomic detection of the *petB* marker gene for *Synechococcus* clade identification

In addition to the metabarcoding approach applied to the three monitoring stations, a total of 14 metagenomes were generated from Station B (Fig. 1). This metagenomic dataset was specifically used to detect the *petB* marker gene and track the *Synechococcus* lineages inhabiting the lagoon. Metagenomic reads were processed and annotated as described below.

Raw metagenomic reads (~ 36–54 million reads per sample) were trimmed with TRIMMOMMATIC v0.39 [40] using the following parameters to remove low quality reads: LEADING:3 TRAILING:3 SLIDINGWINDOW:4:15 MINLEN:50. Contig assembly was performed individually on each sample using MEGAHIT v1.2.9 [41] with meta-large preset and a minimum contig length of 500 bp. Prokaryotic protein coding regions were predicted from the contigs with PRODIGAL v2.6.3 [42]. Predicted protein sequences longer than 100 bp were kept and clustered at 95% identity using CD-HIT v4.8.1 [43] with 90% alignment coverage and 80% length difference cutoff (-c 0.95 -G 0 -aS 0.9 -r 1 -g 1 -d 0 -s 0.8). A final non-redundant dataset of 6,817,971 gene clusters was obtained. The non-redundant gene catalog was screened for functional annotation to orthologous groups (OGs) with eggNOG-mapper v2.1.6 by calling Diamond [44] in sensitive mode and using default options (–itype CDS –translate –sensitive –evalue 0.001) [45, 46]. A total of 4,617,413 genes were functionally annotated. The eggNOG-based functional annotation includes a KEGG (Kyoto Encyclopedia of Genes and Genomes)-based annotation (KOs) for each OG, and was used to search for the *petB* marker gene KO (K02635) in our gene catalog. This gene encodes a subunit of the cytochrome b6f complex in cyanobacteria and other photosynthetic organisms. A total of 106 genes were assigned to K02635, and according to the taxonomic classification provided by eggNOG, 36 of them were identified as cyanobacterial (median annotation score: 331; median *e*-value: 1e^−114^), while the remaining sequences were attributed to eukaryotic organisms. Nucleotide sequences of genes annotated to *petB* KO were taxonomically assigned using BLASTn (BLAST + v2.15.0; options -task megablast -perc_identity 94 -max_target_seqs 10 -*e*-value 0.001) [47] against the NCBI’s non-redundant (nr) database (April 2024) at 94% identity, a cutoff value proposed to provide an optimal separation of *Synechococcus* lineages [48, 49]. Final taxonomic assignment was manually curated based on alignment length and query coverage of the top 10 BLAST hits per query.

Finally, to estimate the abundance of each gene in each sample, cleaned reads from each sample were back-mapped to the non-redundant gene catalog using Bowtie2 v2.2.9 [50] using default options, keeping only mapping hits with quality score greater than 10 with SAMtools v.1.13 (options -F 260 -q 10) [51]. The abundance of the non-redundant gene clusters for each metagenome was retrieved with HTSeq v1.99.2 using function *htseq-count* [52] and normalized by gene length using an in-house script. After normalization, a total of 23 cyanobacterial *petB* genes out of the 36 initially detected were retained.

### Integrated analysis of community structure and environmental drivers

All statistical analyses on environmental factors and community data analysis were performed in R v4.2.0 (R Core Team 2022) [53]. The seasonality of environmental data was assessed using the Lomb-Scargle periodogram (R package ‘lomb’). Specifically, we tested for seasonal patterns in temperature, irradiance, salinity, pH, oxygen, nutrient concentrations, Chla *a*, and the abundance of prokaryotes (*Synechococcus* and heterotrophic bacteria). A variable was classified as seasonal when its maximum normalized power exceeded 5. An annual cycle was inferred when the normalized power at the 1‑year period was greater than 3.

Community composition analyses based on 16S rRNA gene ASVs were managed using the ‘phyloseq’ [54] and ‘tidyverse’ [55] packages. Relative abundance data normality was assessed applying the Shapiro–Wilk test (*shapiro.test* function from base R). Since normality assumptions were not met, differences in community composition among sampling stations were evaluated using the non-parametric Kruskal–Wallis test (*kruskal.test* function from base R). Alpha diversity metrics were calculated with the *estimate_richness* function from the ‘phyloseq’ package. To test for seasonal patterns of prokaryotic communities, a PERMANOVA test was performed (*adonis2* function) on the Bray–Curtis distance matrix.

For ASV-based community structure analyses, Bray–Curtis dissimilarities were computed using the *ordinate* function from the ‘phyloseq’ package and the resulting distance matrices were first visualized through Non-metric MultiDimensional Scaling (NMDS) plots. Then, in order to assess the percentage of variability explained by environmental variables on the prokaryotic community structure, a distance-based redundancy analysis (dbRDA) was performed using the *capscale* function base on Bray–Curtis dissimilarities. Prior to ordination, environmental variables were log(x + 1) transformed and standardized (z-score) using base R functions. An initial dbRDA model including all environmental variables was fitted, and multicollinearity among predictors was assessed by calculating variance inflation factors (VIFs) with the *vif.cca* function. Only variables showing low collinearity (VIF < 10) were retained for the final dbRDA model. The explanatory power of the final model was evaluated using adjusted R^2^ calculated with the *RsquareAdj* function. The overall significance of the dbRDA model and of individual explanatory variables was assessed using permutation tests (999 permutations) implemented with the *anova* function. Significant explanatory variables (*p* < 0.01) were visualized in an dbRDA plot. Additionally, to evaluate the conditional importance of environmental variables, a forward selection procedure was performed using the *ordiR2step* function (999 permutations), where variables were added sequentially according to their contribution to the explained variance in the dbRDA model. All referred functions for multivariate analyses and permutation tests were implemented in the ‘vegan’ R package [56].

Finally, pairwise correlations between the abundance of sequence variants (ASVs and *petB* gene) and environmental variables were assessed using the Pearson correlation coefficient, implemented via the *rcorr* function from the ‘Hmisc’ package [57], with Bonferroni correction applied. Prior to the correlation analyses, *petB* abundance data were transformed using several approaches (centered log-ratio [CLR], log(x + 1), square-root, and arcsine square-root) to evaluate the robustness of associations across different data normalization strategies. All data visualizations were generated using ‘ggplot2’ [58].

### Reconstruction of *Synechococcus* metagenome-assembled genomes and abundance estimation

To reconstruct *Synechococcus* metagenome-assembled genomes (MAGs), metagenomic samples were pooled in two sets based on the relative abundance of *Synechococcus* in the 16S rRNA gene sequence data. The first co-assembly (coass-1) included metagenomes from October 2019 and February 2020 (2 samples), and the second co-assembly (coass-2) was generated from metagenomes collected between July 2021 and October 2022 (7 samples). A previous attempt to reconstruct *Synechococcus* MAGs using a co-assembly of all samples did not yield bins of sufficient quality to meet our criteria (details not shown). For MAG reconstruction, raw reads from the metagenomes were trimmed and cleaned using fastp v0.20.0 [59] with the following parameters: -q 30 -l 25 –detect_adapter_for_pe –trim_poly_g –trim_poly_x. The trimmed reads were assembled into contigs with MEGAHIT v1.1.2 [41] using default settings. Reads were mapped against the contigs using Bowtie2 v2.4.5 [50] with default options, and the resulting .sam files were converted to .bam files using the *samtools* function in the SAMtools v1.9 software [51]. The function jgi_summarize_bam_contig_depths from Metabat2 v2.12.1 [60] was used to estimate the coverage values of each contig of the co-assemblies. Bins were then generated with the function *metabat2* from the same software using default options. The completeness, contamination, and strain heterogeneity of each bin were assessed with CheckM v1.1.2 [61] using the taxonomy_wf life Prokaryote workflow. All bins were taxonomically classified using GTDB-Tk v1.5.0 [62] with the *classify_wf* workflow against the Genome Taxonomy Database (GTDB; release 214). A total of 6 and 9 bins assigned to *Synechococcus* were recovered from coass-1 and coass-2, respectively. A total of 5 MAG bins, each presenting a completeness above 75%, were considered for further analyses.

The abundance of MAGs in each metagenomic sample was calculated by using a command-line workflow available at https://github.com/felipehcoutinho/virathon. Briefly, Bowtie2 was first used to build a reference database containing the contigs associated with the MAGs (MAGsDB). Quality-filtered metagenomic reads from each metagenome were then mapped against the MAGsDB using Bowtie2 in sensitive mode. The resulting .sam files were converted to .bam format and sorted using SAMtools, yielding a raw contig-level abundance table. Contig abundances were subsequently normalized by contig length using base R functions. Finally, MAG abundance in each metagenomic sample was calculated as the sum of the length-normalized read counts of all contigs assigned to the corresponding MAG.

### Functional analysis of *Synechococcus* MAGs and related cultured species

In order to evaluate the functional capabilities that may explain the dynamics of the main *Synechococcus* variants detected in the lagoon, we examined the presence or absence of a custom list of ecologically significant genes in reconstructed *Synechococcus* MAGs from our metagenomes. To achieve this, we first searched for differences in gene content within the reference genomes of the corresponding cultured species to which the MAGs were taxonomically assigned. The information on gene presence/absence for these cultured species was obtained from a gene matrix previously reported by Cabello-Yeves and colleagues [63], which includes data from 123 culture-derived marine, brackish, and freshwater picocyanobacterial genomes (see Table S2 in the aforementioned publication). From this matrix, we identified a list of genes uniquely associated with the cultured species related to the dominant *Synechococcus* variant in our dataset, providing a preliminary set of candidate functions for further filtering and interpretation (summarized in Table S6).

In addition, the reference genomes of the cultured *Synechococcus* species (downloaded from either NCBI-Genbank non-redundant database or the Cyanorak database) were also functionally annotated to KOs [64] using Anvi'o (anvi-estimate-metabolism -output-modes hits) [65, 66]. A tutorial for the modes of usage and input/output files formats to run this program can be found at https://anvio.org/tutorials/fmt-mag-metabolism/. This additional functional annotation allowed us to: (i) curate the initial list of genes obtained from the gene matrix by discarding gene homologs or functional redundancy (i.e., genes with the same KO but listed as separate entries in the gene matrix) and (ii) identify the presence/absence of a few KOs of interest related to genes not listed in the work of Cabello-Yeves and colleagues [63].

Considering the initial list of unique genes and the results of the functional annotation with Anvi'o, a final list of 16 genes considered ecologically relevant was compiled (summarized in Table 2). The presence of these genes in our *Synechococcus* MAGs was searched by building profile Hidden Markov Models (HMMs) for each one. Briefly, amino acid sequences for each gene, sourced from the phylum Cyanobacteria (and, if needed, from other bacterial phyla) were dereplicated at 90% of amino acid identity with CD-HIT v.4.8.1 [43] and aligned using MAFFT v.7.475 [67]. The HMM profiles were then built from those alignments using HMMER v.3.3.2 (http://hmmer.org/). The *Synechococcus-*assigned MAG sequence dataset was used as input for open reading frame (ORF) prediction using the metagenomic mode in Prodigal v.2.3.6 [42]. The 16 HMMs were searched against the amino acid sequences encoded by the predicted ORFs using HMMER with a bitscore cutoff of 50. Functional annotation of the putative hits was further validated by running a BLASTp against the NCBI non-redundant database with an *e*-value cutoff of 1e^−20^.

In addition, because phage defense systems have been linked to cyanobacterial bloom dynamics [33], we conducted a comprehensive search for antiviral defense genes in the MAGs as well as in publicly available genomes of related species using two complementary online servers: DefenseFinder (https://defensefinder.mdmlab.fr/) [68–70] and PADLOC (https://padloc.otago.ac.nz/padloc/) [71, 72]. Even though PADLOC accounts for clustered regularly interspaced short palindromic repeat (CRISPR)-CRISPR-associated protein (Cas) genes, we conducted an additional search with CRISPRCasTyper [73].

### Virus identification, taxonomy, host prediction, and abundance estimation

To explore the role of biotic interactions in regulating cyanobacterial blooms, we examined the temporal dynamics of phages in the lagoon, with a particular emphasis on *Synechococcus*-infecting phages. Contig sequences larger than 5 kb from the 14 metagenomes were searched for double-stranded DNA and single-stranded DNA viruses infecting prokaryotes, following the protocol recommended by Guo and colleagues [74]. Briefly, candidate viral contigs were identified using VirSorter2 v.2.2.4 [74] with a permissive minimum score threshold of 0.5 to maximize sensitivity, and later processed with CheckV v.1.5 [75] to remove non-viral contigs and trim prophage host regions. Retained contigs longer than 5 kb were functionally annotated using DRAM v1.4.6 [76]. To further reduce false positives, we manually inspected contigs carrying genes commonly found in both viruses and their hosts. The viral contig sequences were then clustered into viral operational taxonomic units (vOTUs), approximately equivalent to “populations” or “species-level” taxa of viruses (sensu Gregory and colleagues [77]) by using MUMmer v3.23 [78] with 95% of average nucleotide identity and 80% of alignment fraction of the smallest sequence.

Virus-host predictions were performed using the software iPHoP v.1.3.3 [79] and the database version iPHoP_db_Aug23_rw. Viruses were assigned to *Synechococcus* hosts following the GTDB (Genome Taxonomy Database) classification [80] if predictions showed a confidence score of ≥ 90.

To estimate the abundance of phages, quality-trimmed metagenomic reads were mapped to the representative contigs of all vOTUs using CoverM v.0.7.0 [81]. Only reads mapping with ≥ 95% of nucleotide identity, covered over ≥ 70% of their length and aligned to contig sequences with ≥ 75% coverage were retained. Coverage values were subsequently normalized to the total number of base pairs in the corresponding metagenome to obtain the final abundances (read coverage per metagenomic Gb). To measure the enrichment of viral communities in phages putatively infecting *Synechococcus* over time, we divided the sum of relative abundances derived from vOTUs assigned to this host by the total abundance of all vOTUs in each metagenome. Alpha diversity metrics (Chao1 richness and Pilou’s evenness) of *Synechococcus*-infecting phage communities were calculated using functions *diversity, specnumber* and *estimateR* from the ‘vegan’ R package [56]. Moreover, to explore the temporal taxonomic succession of viruses infecting *Synechococcus*, representative contigs from vOTUs longer than 10 kb were taxonomically classified using vConTACT3 (v3.1.6) [82] with the prokaryotic reference database (v230). Abundances of these vOTUs were aggregated according to the lowest available taxonomic rank at or above the family level. For each sample, the summed abundance of vOTUs associated with a given viral taxon was expressed as a percentage of the total abundance of all vOTUs. These values were visualized as a heatmap using the R package ‘pheatmap’ (v1.0.13) [83].

## Results

### Temporal variability of biotic and abiotic factors

Our study spans a period of three years, from October 2019 to October 2022, during which DNA sampling was integrated into the existing monitoring program of the Spanish Institute of Oceanography that began in June 2016, at three stations in the lagoon (Fig. 1). The environmental data collected since the beginning of the monitoring period (from June 2016 to October 2022) are shown in Figs. S1, S2, and S3. Values represent averages across the three stations (A, B, and C), which generally exhibited similar patterns, with some exceptions reflected in the standard deviation shown in the graphs. The Lomb-Scargle periodogram revealed that certain variables exhibited a clear seasonal pattern with a periodicity of one year (Table S1). The strongest seasonal signal corresponded to water temperature, which ranged from minimum mean values in January (12.2 ± 1.3 °C) to maximum mean values in July–August (29.3 ± 0.9 °C). Salinity and dissolved oxygen also displayed annual periodicity, although their characteristic values were occasionally deviated from the expected pattern (Fig. S1). Among nutrients, nitrate and silicate showed consistent annual cycles (Fig. S2; Table S1). In contrast, no seasonal signal was detected for the remaining nutrients, or for pH, light extinction coefficients (K_d_), chlorophyll *a* concentrations, or prokaryotic cell abundances (Fig. S3)*.* The first DNA sampling took place in October 2019 during a deoxygenation event that followed an intense rainfall episode characteristic of the Spanish Mediterranean coast known as ‘cold drop’, which occurred between 11 and 15 September 2019. During this event, a diatom bloom was reported [27] and oxygen levels dropped in the three stations (< 1 mg L^−1^ at 6 m depth and around 5 mg L^−1^ at 4 m depth) (Fig. S1), while mean Chl *a* concentration reached 38.5 ± 27.1 µg L^−1^, the highest value registered since the monitoring started in June 2016 (and up to 66 µg L^−1^ in station B) (Fig. S3). Consequently, the light extinction coefficient (K_d_) increased in the water column (up to 1.35 m^−1^), reducing photosynthetically active radiation (PAR) at the bottom to less than 0.5% (Fig. S1). Overall, after this intense rainfall event, the salinity in the lagoon dropped from a mean value of 44.4 ± 1.0 in previous years to a mean value of 41.3 ± 1.5 in subsequent years (October 2019 to October 2022), with the lowest salinity observed around February/March 2020 (38.3 ± 0.22). A second deoxygenation event occurred in August 2021, also linked to diatom proliferation [27], during which oxygen concentrations hovered around or below the hypoxia threshold (5 mg L^−1^). This oxygen decline was accompanied by an increase in K_d_ and by another peak in Chl *a* concentration (ca. 22 µg L^−1^ at stations B and C). Salinity remained stable during this second event. Besides these two deoxygenation events, Chl *a* concentration in the lagoon from 2019 onwards generally remained below 2 µg L^−1^ (75th percentile).

Since the beginning of the lagoon's monitoring, several peaks in the abundance of the cyanobacterium *Synechococcus* have been recorded (Fig. 2 and S3). An increase in its abundance occurred immediately following the autumn 2019 ‘cold drop’, with mean cell abundances reaching 4.4 × 10^5^ cells mL^−1^ in October, when the first DNA sampling was conducted, and peaking at 1.56 × 10^6^ cell mL^−1^ in December 2019 (an 18-fold increase compared to the mean value of the preceding months in 2019). A continuous decline followed, with *Synechococcus* abundances decreasing to approximately 5 × 10^5^ cell mL^−1^ by April 2020 (Fig. 2). Another pronounced peak (1.4 × 10^6^ cell mL^−1^; a 60-fold increase relative to the preceding months in 2021) was detected on 19 August 2021, coinciding with the second deoxygenation event. However, cell numbers dropped rapidly thereafter, reaching approximately 10^4^ cell mL^−1^ by 26 August 2021. Aside from the August 2021 peak, *Synechococcus* abundances remained comparatively low after April 2020, with a median of 3 × 10^4^ cell mL^−1^ for the rest of the time series and minimum values around 10^3^ cell mL^−1^. Heterotrophic bacterial abundances during the study period ranged between 3 × 10^5^ and 9.8 × 10^6^ cell mL^−1^ (median: 2.4 × 10^6^ cell mL^−1^) and generally increased following *Synechococcus* peaks, although additional bacterial maxima occurred sporadically throughout the time series (Figs. 2 and S3).

### Prokaryotic community composition and structure

**Fig. 3:**
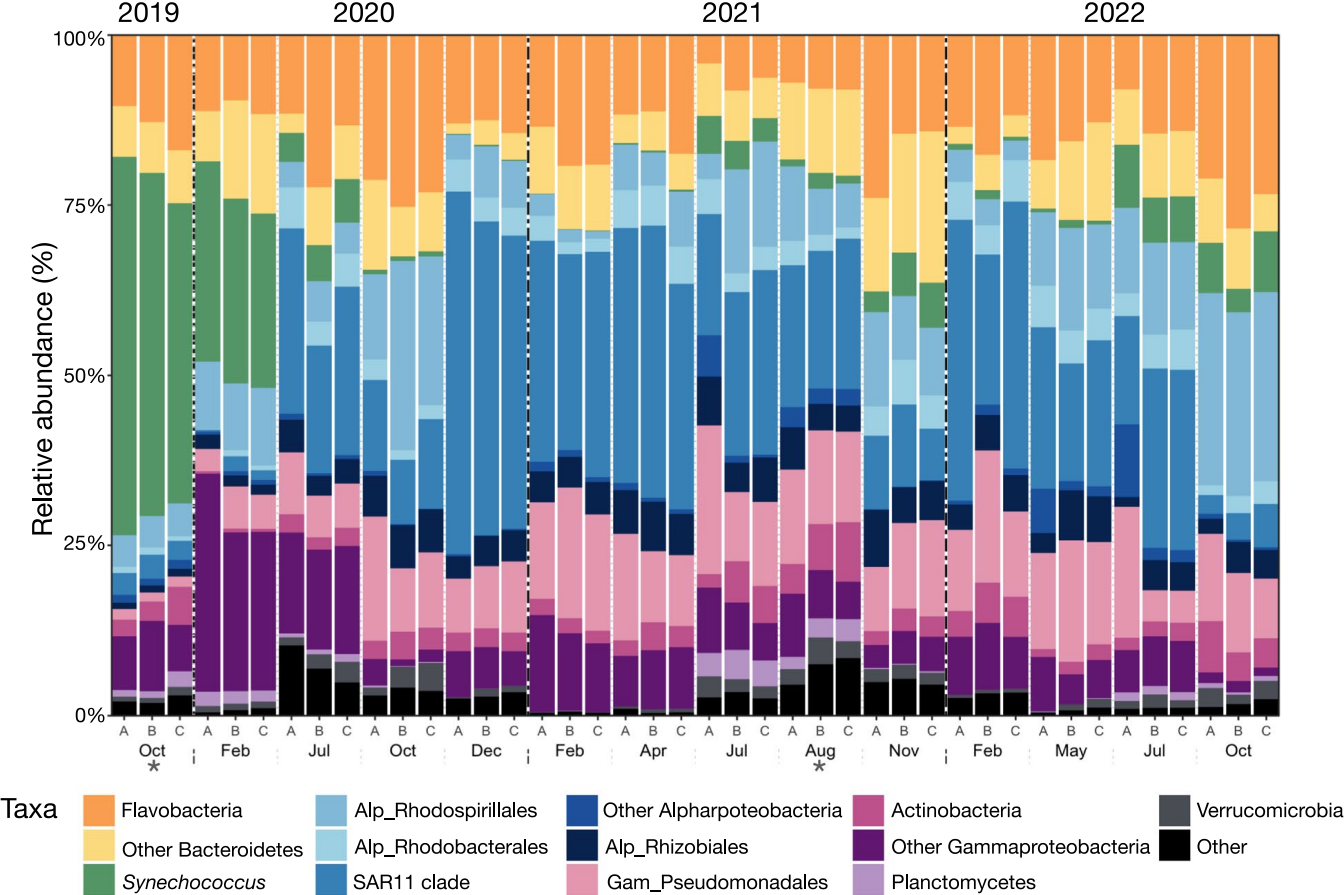
Bar plot showing the relative abundance of the main prokaryotic taxa over the 3-year study period. For each sampling date, data from stations A, B, and C, are shown, as indicated in the x-axis. Asterisks mark the dates on which the two deoxygenation events occurred. The sampling year is displayed above the panel. Taxonomic groups are color-coded, with the legend shown at the bottom. ASVs not affiliated with any of the main taxa were grouped as ‘Other’. Alp: Alphaproteobacteria; Gam: Gammaproteobacteria

Based on the 16S rRNA gene sequencing data, the composition of the prokaryotic community indicated that, (i) there were no significant differences among sampling stations (Kruskal–Wallis *t*-test non-significant; *p* > 0.05); and (ii) the relative contributions of the main groups changed over the study period, with a notable shift in the relative abundance of cyanobacteria compared with heterotrophic bacterial taxa (Fig. 3).

*Synechococcus* was dominant following the intense rainfall event in autumn of 2019 (mean ca. 50% across the three stations) and remained abundant until February 2020 (Fig. 3). Afterwards, *Synechococcus* virtually disappeared for several months, reappearing only at low proportions (< 10%) by July 2021, and persisted at low levels for the rest of the study period. The relative abundance of Actinobacteria ASVs was high in February 2020 (mean ca. 26%), just before the decline of *Synechococcus*. The predominant Actinobacteria variants (ASV_6 and ASV_18) reached relative abundances exceeding 15% each in February 2020. Following the decline in *Synechococcus*, several taxa within the Alphaproteobacteria, particularly the marine SAR11 clade and Rhodobacterales, as well as members of the Gammaproteobacteria, particularly Pseudomonadales*,* dominated the assemblages for the rest of the time series. Indeed, some of the most abundant ASVs in our dataset (ASV_1 and ASV_4) were affiliated with the SAR11 clade, each reaching up to 26% of the total reads. Members of the Flavobacteria and other Bacteroidetes were prevalent throughout the entire study period, ranging from approximately 12% to 38% in relative abundance. In general, Archaea were present at very low abundances (< 1%) and were grouped under the category “Others”. Interestingly, the prokaryotic composition of samples collected around the second deoxygenation event in August 2021 did not show marked differences from other summer samples in the dataset and was dominated by members of the Alpha‑ and Gammaproteobacteria (Fig. 3).

**Fig. 4:**
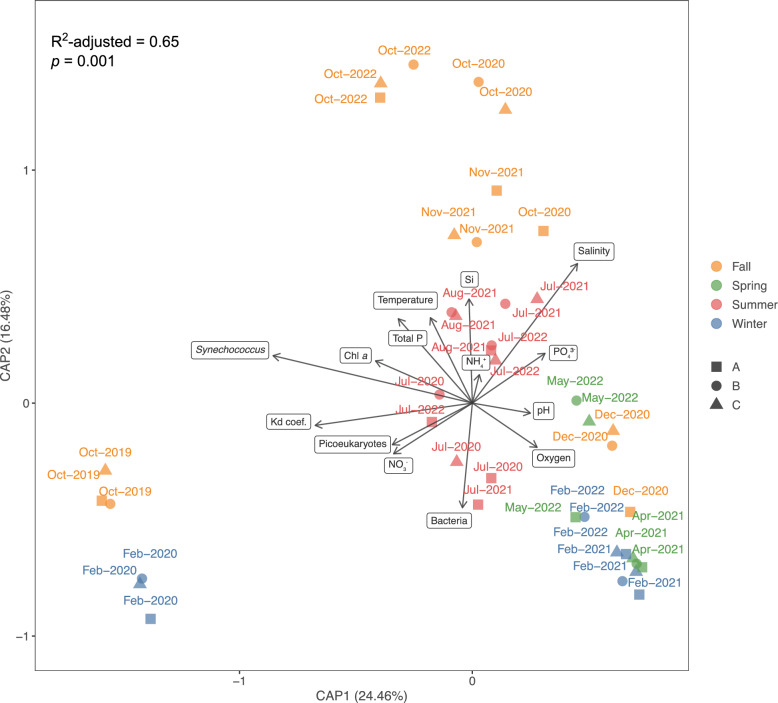
Distance-based redundancy analysis (dbRDA) ordination illustrating the similarity of prokaryotic communities in samples collected between October 2019 and October 2022. Samples are color-coded by season and differentiated by sampling station through symbol shape. Arrows represent environmental and biological variables that significantly explained community variation (*p* < 0.01); arrow length indicates the strength of the relationship with the ordination axes. Percentages on the axes indicate the proportion of constrained variation explained. The adjusted R^2^ and *p*-value of the dbRDA model are shown

No clear seasonal trends in alpha diversity were observed (Fig. S4). However, lower values of the Shannon and Simpson diversity indices were recorded at the beginning of our study, coinciding with the first deoxygenation event (October 2019). In contrast, higher values of the Chao1 richness index and the Shannon and Simpson indices coincided with the second deoxygenation event (August 2021). Regarding beta diversity, the structure of the prokaryotic community exhibited a weak but significant seasonal pattern (Fig. S5A) (PERMANOVA, season R^2^ = 0.247, *p* < 0.001). To assess the influence of environmental variables on community composition, a distance-based redundancy analysis (dbRDA) was performed (Fig. 4). The dbRDA ordination indicated that community structure was strongly organized along environmental and biological gradients rather than by season alone. The dbRDA model including all non-collinear predictors explained 65% of the variation in community structure (adjusted R^2^ = 0.65) and was highly significant based on permutation tests (F = 6.40, *p* = 0.001). The first two constrained axes explained 24.5% and 16.5% of the constrained variation, respectively. CAP1 primarily reflected a gradient opposing *Synechococcus* abundance, light attenuation (K_d_), Chl *a*, and picoeukaryote abundance to higher salinity and phosphate concentrations. CAP2 separated samples according to temperature and nutrient availability versus oxygen. Samples collected during cooler periods (December to May) clustered towards negative CAP2 values and were associated with higher oxygen concentrations and lower temperature and Chl *a* values. Samples from warmer periods (i.e. August and October) shifted toward positive CAP2 values and were associated with higher temperatures and lower heterotrophic bacterial abundances. In addition, the analysis revealed a clear separation of samples from October 2019 and February 2020 from the rest of the dataset along CAP1. This separation was primarily driven by higher K_d_, increased *Synechococcus* abundance, and reduced salinity, consistent with the impact of the ‘cold drop’ affecting the lagoon (see Fig. S5B). When these two post- ‘cold drop’ samples were removed, the PERMANOVA R^2^ value increased (R^2^ = 0.342, *p* < 0.001), indicating that the seasonal signal would be stronger in the absence of extreme disturbance events.

To further assess the relative importance of environmental and biological drivers structuring the community, a forward-selection procedure was applied to the dbRDA model, ranking variables according to their sequential contribution to the explained variance. Permutation tests based on the conditional effects of each predictor revealed that *Synechococcus* abundance and salinity were the strongest contributors (F = 21.87, *p* = 0.001; F = 12.59, *p* = 0.001, respectively), followed by heterotrophic bacterial abundance (F = 8.93, *p* = 0.001), temperature (F = 8.34, *p* = 0.001), and Chl *a* (F = 6.66, *p* = 0.001). The remaining variables also showed significant effects (*p* < 0.05), although their contributions were progressively smaller. In contrast, dissolved oxygen, total P, and phosphate concentrations did not explain additional variation in community structure and were therefore not retained as significant predictors (Table S2).

### Characterization of the *Synechococcus* assemblages

**Fig. 5:**
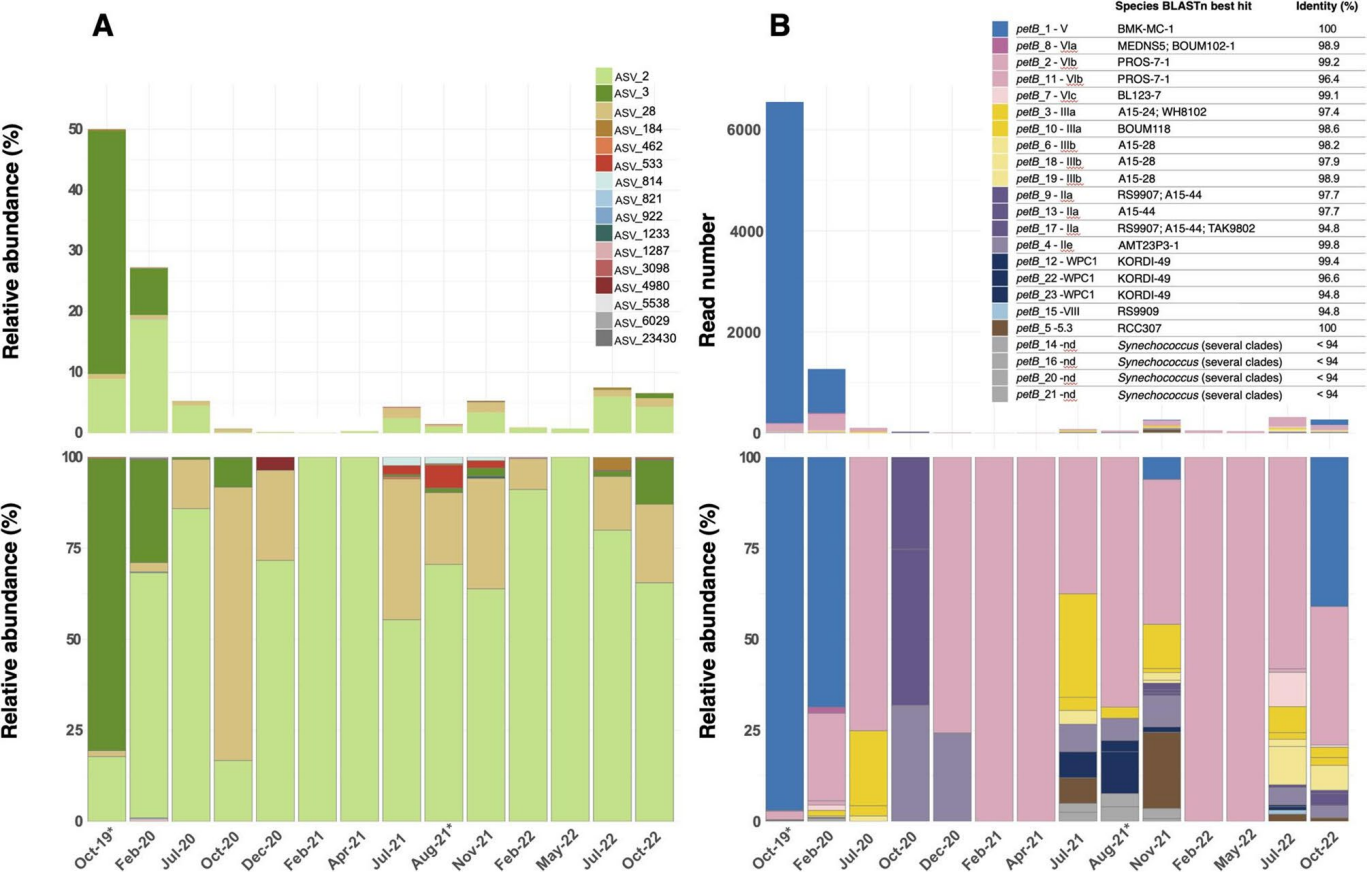
Temporal dynamics of *Synechococcus* sequence variants. (**A**) Relative abundance of *Synechococcus* 16S rRNA gene amplicon sequence variants (ASVs), shown as the proportion of total prokaryotic reads (top panel) and as the proportion of total *Synechococcus* reads (bottom panel). Values represent means from stations A, B, and C. (**B**) Relative abundance of *Synechococcus petB* gene sequence variants over time for Station B. The 23 detected variants are listed in the accompanying table, color-coded according to their assigned subclades (subclade numbers indicated after the gene number). The best BLASTn hit and percent identity are also shown; nd = subclade not determined. The top panel shows total metagenomic *petB* read counts (normalized by gene length), while the bottom panel depicts the relative contribution of each variant to total *petB* reads assigned to *Synechococcus*. In both panels, asterisks mark the dates on which the two deoxygenation events occurred

Given the reported importance of *Synechococcus* in the onset of the initial EDAB in 2016 and the abundance shifts observed over our three-year study period, we further examined its population dynamics by analyzing the distribution of its specific ASVs in the lagoon (Fig. 5A). We detected 16 *Synechococcus*-related ASVs, most of which differed by only one or two nucleotide mismatches. The *Synechococcus* assemblages were dominated by three alternating ASVs, each differing by a single nucleotide. In October 2019, ASV_3 dominated the *Synechococcus* populations (~ 75%) and remained well represented in February 2020 (~ 25%). However, ASV_3 virtually disappeared from subsequent samples, occurring only occasionally at proportions below 10%. In contrast, ASV_2 became prominent in February 2020 (~ 75%) and dominated the *Synechococcus* populations for the rest of the study period, except in October 2020 when ASV_28 was dominant. Notably, ASV_28 consistently appeared during summer (July, August) and fall (October, November, December) months. Potential correlations between the relative abundances of *Synechococcus* variants and environmental variables were evaluated (Fig. S6). ASV_3 proportions were positively correlated with the light extinction coefficient values (Pearson’s R = 0.79, *p* < 0.05). ASV_2 proportions showed a positive correlation with nitrate concentrations (R = 0.68, *p* < 0.05) and a negative correlation with salinity (R = − 0.60, *p* < 0.05), as well as other minor variants in the dataset. No significant correlation was found for ASV_28, the third most abundant variant.

To better understand the *Synechococcus* dynamics, we aimed to determine whether these variants represented populations with distinct functional traits that could explain their temporal alternation. These ASVs were initially taxonomically assigned to *Synechococcus* using the SILVA database. For higher taxonomic resolution, we compared their classification against the Cyanorak database [39] (Table S3), which includes information on complete genomes of *Synechococcus* species and their classification into phylogenetic clades based on the functional gene *petB*, a high-resolution taxonomic marker widely used to effectively differentiate ecologically relevant *Synechococcus* lineages [48, 49]. The dominant *Synechococcus* ASV in October 2019 (ASV_3) exhibited 100% identity with the 16S rRNA gene sequences of cultured isolates BMK-MC-1 and WH7803, both belonging to *Synechococcus* subclade V. The second most abundant ASV (ASV_2) also showed 100% identity, but with several *Synechococcus* species distributed across multiple subclades (VIa, VIb, VIIa). Likewise, ASV_28, the third most abundant variant, showed 100% identity with species distributed across subclades IIa, IIc, IIh, IIIa, and IIIb. These results confirmed that, as expected, the 16S rRNA gene lacked the necessary resolution to accurately differentiate between *Synechococcus* clades.

To overcome this limitation, we investigated the functional *petB* gene using metagenomic data from Station B. In total, 23 cyanobacterial *petB* gene sequences were detected, most of which (n = 19) were closely related or identical to known *Synechococcus* species (> 94% identity) and could be classified into 11 distinct *Synechococcus* subclades according to the framework established by Farrant and colleagues [49] (Table S4). The remaining sequences (n = 4) exhibited < 94% similarity to reference *Synechococcus* sequences, below the established threshold for defining *petB* clades [48, 49], and therefore, could not be confidently assigned to a specific clade. Among the classified sequences, all but one belonged to subclades within the large subcluster 5.1, which comprises mostly obligate marine species; the remaining sequence was affiliated with subcluster 5.3, which includes both marine and freshwater representatives [63]. As performed for the 16S rRNA gene *Synechococcus* ASVs, we analyzed the temporal dynamics of *petB* gene variants across the time series (Fig. 5B). The overall abundance patterns of *petB* closely mirrored those of *Synechococcus* 16S rRNA gene reads (Fig. 5B). The highest *petB* read abundances (> 10^3^) were detected at the beginning of the time series (October 2019 and February 2020), followed by a decrease of approximately two orders of magnitude in most subsequent samples. Consistent with the 16S rRNA gene results, two main *petB* variants dominated the *Synechococcus* assemblages. Although the dominant 16S rRNA gene ASVs (ASV_2 and ASV_3) showed significant associations with environmental variables (Fig. S6), none of the *petB* variants displayed significant correlations under any of the normalization strategies tested.

The two dominant *petB* variants correspond to distinct *Synechococcus* subclades. The most abundant variant, *petB*_1 (shown in blue in Fig. 5B), displayed 100% identity with the *petB* sequence from the isolate *Synechococcus* sp. BMK-MC-1, which belongs to subclade V, while the second most abundant variant, *petB*_2 (shown in pink in Fig. 5B), exhibited 99.2% identity with *Synechococcus* sp. PROS-7-1 from subclade VIb. Both variants mirrored the abundance patterns observed for the 16S rRNA gene variants ASV_3 and ASV_2, respectively. The third most abundant variant, *petB*_3 (shown in yellow in Fig. 5B), showed a lower read abundance –about an order of magnitude lower (Table S4)– and was assigned to subclade IIIa with 97.4% similarity to the cultured isolate *Synechococcus* WH8102. Its abundance pattern largely mirrored that of the 16S rRNA gene ASV_28, except in October 2020, and it consistently appeared in July 2020, July 2021, and July 2022, as well as in November 2021 and October 2022. Interestingly, in October 2020, *Synechococcus* assemblages were almost entirely composed of variants related to subclade II (shown in purple in Fig. 5B). The most abundant among these, *petB*_4 (shown in light purple in Fig. 5B) showed 99.8% similarity to the environmental sequence AMT23P3-1, assigned to subclade IIe reported in Farrant and colleagues [49]. To facilitate comparisons of *Synechococcus* variants detected using different approaches, the potential associations between 16S rRNA gene variants, *petB* gene variants, MAG bins and *Synechococcus* sp. isolates are summarized in Table 1.

**Table 1:**
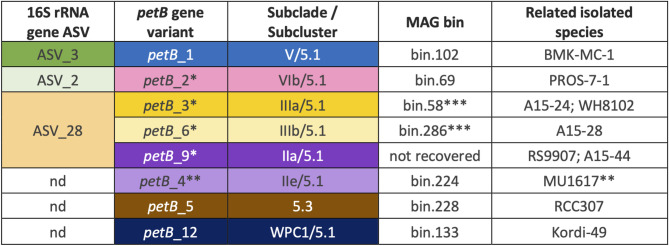
Correspondence among the *Synechococcus* marker-gene variants (16S rRNA and *petB*) and the reconstructed MAG bins from the Mar Menor, along with their closest related isolated species

*For simplicity, in subclades with more than one assigned *petB* variant, only the most abundant variant is listed; for the remaining minor variants assigned to the same subclades see Fig. 5B or Table S2

**petB_4 variant was initially assigned with BLASTn to the *petB* environmental sequence AMT23P3-1; this environmental sequence shows 99.5% similarity to the *petB* sequence extracted from the environmental genome MU1617

***Low completeness bin

nd: not determined

### Functional traits of *Synechococcus* populations

After examining the temporal distribution patterns of *Synechococcus* genetic variants, we sought to determine whether shifts in their populations reflect differences in functional capabilities, such as tolerance to specific stressors or resource utilization preferences. To address this, we reconstructed *Synechococcus* metagenome-assembled genomes (MAGs) from our lagoon metagenomes and assessed the presence or absence of ecologically relevant genes. Because MAG completeness can influence the accuracy and depth of functional profiling [84] we first examined the gene content of reference genomes from cultured *Synechococcus* species closely related to the Mar Menor MAGs. A total of 15 bins (i.e., MAGs) assigned to *Synechococcus* were recovered from the metagenomes: six from the co-assembly of samples collected in October 2019 and February 2020 (coass-1) and nine from the co-assembly of samples collected between July 2021 and October 2022 (coass-2) (Table S5). Among the recovered *Synechococcus* MAGs, five were selected for functional analysis, each with an estimated completeness above 75% (bins 69, 102, 133, 224, and 228). The abundance pattern of these MAGs was evaluated across the time series (Fig. S7). MAG bin.102 was dominant in the aftermath of the intense rainfall event of autumn 2019, and exhibited read abundances one order of magnitude higher (> 10^5^) in October 2019 and February 2020 than in subsequent sampling dates. In contrast, MAG bin.69 replaced bin.102 from February 2020 onwards, remaining the dominant MAG –although at lower abundance– for the rest of the study period. The temporal dynamics of these two MAGs closely matched those of the corresponding *petB* variants, *petB*_1 and *petB*_2, respectively. The contribution of the remaining MAG bins was minor throughout the time series.

MAG bin.102, reconstructed from coass-1, was assigned to *Synechococcus* sp. WH7803, a representative species in GTDB, with an average nucleotide identity (ANI) of 97%. However, the *petB* sequence extracted from this MAG showed higher similarity to the closely related species BMK-MC-1 (99.9% identity; one mismatch) than to WH7803 (three mismatches). These results are consistent with the sequence similarity of the dominant *petB* variant recovered from these samples, *petB*_1, which was identical to the *petB* sequence of isolate BMK-MC-1 but had 2 mismatches with isolate WH7803. Therefore, the genome of isolate BMK-MC-1 was also considered in the comparative genomic analysis. The remaining four MAGs, generated from coass-2, were taxonomically assigned to *Synechococcus* cultured isolates PROS-7-1 (bin.69), RCC307 (bin.228), KORDI-49 (bin.133), and to the environmental genome MU1617 (bin.224). All of these species were also retrieved in the previous taxonomic assignation of the *petB* sequences from the lagoon (Fig. 5B, Table S4). Specifically, PROS-7-1 corresponded to the second most abundant variant (*petB*_2), RCC307 to variant *petB*_5 and KORDI-49 to variant *petB*_12. The environmental genome MU1617 was closely related to variant *petB*_4 showing 99.4% sequence similarity (see Table 1 for an overview of the correspondence between marker genes, MAG bins and related *Synechococcus* species).

The gene content of the cultured *Synechococcus* species to which our MAGs were most closely related was examined in a presence/absence gene matrix published by Cabello-Yeves and colleagues [63]. This matrix provides a broad perspective on metabolic adaptations of *Synechococcus* to different habitats by containing information on 123 marine, brackish, and freshwater picocyanobacterial cultured isolates (see Material and Methods). From this matrix, we extracted a preliminary list of genes present in species BMK-MC-1 but absent in species PROS-7-1, which are related to our MAG bin.102 and bin.69, respectively (Table S6). After removing redundant genes present in species PROS-7-1, we refined the list to 16 ecologically relevant genes (Table 2), focusing on those involved in nutrient metabolism, transport, and other key ecological pathways. These genes were screened in our MAGs using HMM-based searches (see Materials and Methods). The selected genes were associated with transporters (uptake systems), toxin-antitoxin systems, assimilatory nitrate reduction, and transposases. Notably, these genes were consistently present in the genome of *Synechococcus* species BMK-MC-1 and its related MAG (bin.102), while they were absent in *Synechococcus* species PROS-7-1 and its related MAG (bin.69) (see Table 2). However, the absence of these genes in the MAGs may reflect limitations in genome completeness rather than their true absence in the *Synechococcus* populations in the lagoon.

**Table 2:** Gene-content comparison among representative *Synechococcus* species^1^ (top panel) and among the MAG bins^2^ recovered from the Mar Menor coastal lagoon (bottom panel)

Functional category	Gene	Product	BMK-MC-1	WH7803	PROS-7-1	RCC307	Kordi-49	MU1617
*Transporters*								
Broad transporters / Miscellaneous	*aqpZ*	Aquaporine Z	+	*−*	*−*	*−*	*−*	*−*
Other ATPases / F0F1/V-type ATP-synthases	*evrA*	ATP-binding cassette-type viologen exporter	+	+	*−*	*−*	+	+
	*evrB*	ATP-binding cassette-type viologen exporter, permease component	+	+	*−*	*−*	+	+
	*evrC*	ATP-binding cassette-type viologen exporter	+	+	*−*	*−*	+	+
Nitrate/nitrite transport	*nrtP*	Nitrate transporter	+	+	*−*	+	+	+
*Nitrogen metabolism*								
Assimilatory nitrate reduction	*narB*	Nitrate reductase	+	+	*−*	+	+	+
	*narM*	Nitrate reductase associated protein	+	+	*−*	+	+	+
*Genetic mobile elements*								
Transposases	*insH1*	Transposase and inactivated derivatives, IS5 family	+	*−*	*−*	*−*	*−*	*−*
*Stress response /cellular defense mechanisms*								
Toxin-antitoxin systems	–	Type II toxin-antitoxin system, antitoxin Phd/YefM	+	*−*	*−*	*−*	+	*−*
	–	Putative antitoxin VapB44	+	*−*	*−*	*−*	*−*	*−*
	*vapC*	Type II toxin-antitoxin system VapC family toxin	+	*−*	*−*	*−*	*−*	*−*
	*vapB*	Antitoxin Phd	+	*−*	*−*	*−*	*−*	*−*
	–	YoeB-like toxin of bacterial type II toxin-antitoxin system	+	*−*	*−*	*−*	*−*	*−*
	K07172*	Antitoxin MazE	+	*−*	*−*	*−*	*−*	*−*
	K18843*	Antitoxin HicB	+	*−*	*−*	*−*	*−*	*−*
	K18918*	RHH-type transcriptional regulator, rel operon repressor / antitoxin RelB	+	*−*	*−*	*−*	*−*	*−*

Functional category	Gene	Product	bin.102		bin.69	bin.228	bin.133	bin.224
*Transporters*								
Broad transporters / Miscellaneous	*aqpZ*	Aquaporine Z	+		*−*	*−*	*−*	*−*
Other ATPases / F0F1/V-type ATP-synthases	*evrA*	ATP-binding cassette-type viologen exporter	+		*−*	*−*	*−*	*−*
	*evrB*	ATP-binding cassette-type viologen exporter, permease component	+		*−*	*−*	*−*	*−*
	*evrC*	ATP-binding cassette-type viologen exporter	+		*−*	*−*	*−*	*−*
Nitrate/nitrite transport	*nrtP*	Nitrate transporter	+		*−*	+	+	+
*Nitrogen metabolism*								
Assimilatory nitrate reduction	*narB*	Nitrate reductase	*−*		*−*	+	+	+
	*narM*	Nitrate reductase associated protein	+		*−*	+	+	+
*Genetic mobile elements*								
Transposases	*insH1*	Transposase and inactivated derivatives, IS5 family	*−*		*−*	*−*	*−*	*−*
*Stress response /cellular defense mechanisms*								
Toxin-antitoxin systems	–	Type II toxin-antitoxin system, antitoxin Phd/YefM	+		*−*	*−*	*−*	*−*
	–	Putative antitoxin VapB44	*−*		*−*	*−*	*−*	*−*
	*vapC*	Type II toxin-antitoxin system VapC family toxin	*−*		*−*	*−*	*−*	*−*
	*vapB*	Antitoxin Phd	*−*		*−*	*−*	*−*	*−*
	–	YoeB-like toxin of bacterial type II toxin-antitoxin system	+		*−*	*−*	*−*	*−*
	K07172*	Antitoxin MazE	+		*−*	*−*	*−*	*−*
	K18843*	Antitoxin HicB	+		*−*	*−*	*−*	*−*
	K18918*	RHH-type transcriptional regulator, rel operon repressor / antitoxin RelB	*−*		*−*	*−*	*−*	*−*

^1^Species comparison was based on the gene presence/absence matrix provided in the work of Cabello-Yeves and colleagues [63]. The presence of these genes in the MAGs was assessed using HMM-based searches

^2^MAGs were taxonomically classified according to the species listed above, based on genomic references from GTDB (see Table S5). Note that the gene content information of environmental genome MU1617 was not provided in the work of Cabello-Yeves and colleagues [63] but was added here for comparison with bin.224. For MU1617, gene predictions were performed using the HMMs approach

*Genes not retrieved from the matrix provided by Cabello-Yeves and colleagues [63], but identified via gene prediction using Anvi'o on reference genomes or MAGs sequences

(–) Gene name not assigned

Specifically, the gene encoding aquaporin Z (*aqpZ*) and certain toxin-antitoxin system genes were exclusively detected in the genome of *Synechococcus* isolated species BMK-MC-1 and its associated MAG (bin.102). Interestingly, the *aqpZ* gene was found only in BMK-MC-1 among the 48 marine isolates analyzed by Cabello-Yeves and colleagues [63], despite being widely present in the genomes of freshwater and brackish picocyanobacteria. Similarly, of the 25 toxin-antitoxin system gene entries in the gene matrix, only 18 out of the 48 marine isolates contained at least one such gene. In contrast, these genes were more prevalent in freshwater and brackish species, being present in 65 out of 67 and 14 out of 17 isolates, respectively. For example, marine *Synechococcus* isolated species WH7803, PROS-7-1, and RCC307 showed no hits for any of the toxin-antitoxin genes listed in the matrix. Additionally, the VapBC toxin-antitoxin module –where VapC functions as a ribonuclease toxin and VapB acts as its antitoxin– was detected only in the genome of the *Synechococcus* marine isolate BMK-MC-1 among the 48 isolates analyzed. This module was rarely found in brackish isolates (3 out of 17) and was partially present in freshwater isolates (30 out of 67). Similarly, the *insH1* gene, which encodes a transposase, was detected only in three of the 48 genomes analyzed from marine isolates, including BMK-MC-1, and it appears to be more prevalent in freshwater and brackish isolates.

Among transporters, particular attention should be given to genes *envA*, *envB*, and *envC*, which encode components of an ATP-binding cassette (ABC) exporter for viologen, a nonselective herbicide [85]. This system is widely distributed across picocyanobacteria from various environments, being detected in 64 out of 67 freshwater isolates, 12 out of 17 brackish isolates, and 45 out of 48 marine isolates analyzed in Cabello-Yeves and colleagues [63]. However, it was notably absent in three marine isolated species, including two examined in this study (PROS-7-1 and RCC307). Additionally, other key transporters and enzymes involved in nitrogen metabolism, such as NrtP (Nitrate-Nitrite Transporter) and NarB, NarM (for nitrate assimilation), are commonly found in marine picocyanobacteria (present in 45 out of 48 isolates) but were not detected in the genome of isolate PROS-7-1.

Given that virus-host interactions have been suggested to influence cyanobacterial dynamics [33], we explored the adaptation of *Synechococcus* lineages from the Mar Menor to viral pressures. To this end, we conducted a targeted search for antiviral defense genes across *Synechococcus* MAGs and their closely related cultured species. Bin.102 was found to carry seven different antiviral defense systems: Type I BREX (6 genes), Type IV restriction-modification (RM) (1 gene), Type IIG restriction-modification (1 gene), AbiE proteins associated with abortive infection (2 genes), SanaTA (2 genes), MazEF toxin-antitoxin module (2 genes), and a candidate system (PDC-S44, 1 gene) (Table S7). The genome of isolate BMK-MC-1 encoded the same antiviral systems ─all of them complete, with all the constituting genes─ with slight differences: the absence of Type-IIG RM system, and presence of three additional systems (Type-I and Type-IV restriction endonuclease, CBASS, and PDC-S09). In contrast, the search for antiviral defense systems in the genome of the other MAGs and related isolates revealed only a few hits. *Synechococcus* isolate PROS-7-1 contained two genes of the Type-III RM system, isolate KORDI-49 carried three genes related to DNA modification systems, and a candidate system (PD-T4-6) was identified in bin.133. No antiviral genes were detected in any of the other isolates or MAGs analyzed in this study. Furthermore, none of the *Synechococcus* MAGs contained detectable CRISPR-Cas systems.

### Dynamics of cyanophages in the lagoon

Finally, to assess the role of viruses in top-down control of *Synechococcus* populations, metagenomes were screened for viral sequences and assigned to putative hosts, resulting in the identification of 86 vOTUs predicted to infect this host (Table S8). The relative abundance of these cyanophages to total viral communities over time (Fig. 5) largely mirrored the dynamics of *Synechococcus* populations measured by flow cytometry and 16S rRNA gene metabarcoding (Figs. 2 and 5A): a gradual decline from October 2019 to October 2020, followed by a short period of minimum levels from December 2020 to April 2021, and then four peaks in July 2021, November 2021, July 2022, and October 2022. Also, to explore viral taxonomic succession in response to *Synechococcus* dynamics, we analyzed the temporal trajectories of taxa linked to *Synechococcus*-infecting vOTUs, but no clear or consistent patterns emerged (Fig. S8).

**Fig. 6:**
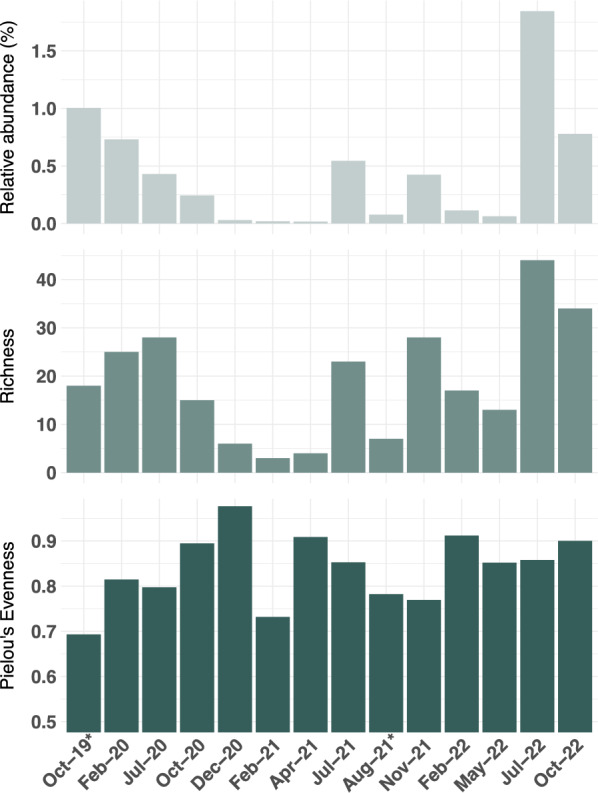
Dynamics of abundance and alpha diversity of *Synechococcus*-infecting phage communities. From top to bottom: percentage of relative abundance of vOTUs predicted to infect *Synechococcus* to total vOTUs, richness (number of *Synechococcus*-infecting vOTUs), and Pielou’s evenness across the 14 time points. Asterisks mark the dates of the two deoxygenation events

Finally, alpha diversity metrics of *Synechococcus*-infecting phages revealed that, despite the high cyanophage abundance in October 2019, this community showed relatively low richness (18 vOTUs) and the lowest evenness (< 0.7) of the time series (Fig. 5). The gradual decrease in abundance until October 2020 matched a reciprocal increase in richness. During the subsequent low-abundance period (December to April 2021), richness dropped to its minimum levels (3 − 6 vOTUs). In contrast, the abundance peaks observed in 2021 and 2022 coincided with higher richness values (> 20 vOTUs).

## Discussion

Previous studies of phytoplankton communities in the Mar Menor revealed that, in 2015, the lagoon experienced an intense cyanobacterial bloom that triggered a drastic ecosystem shift [7]. In the following years (2016–2021), phytoplankton assemblages became dominated by diatoms, which reached unprecedented cell abundances, yet exhibited no clear seasonal pattern [27]. Dinoflagellate abundances similarly showed pronounced interannual variability between 2016 and 2019, at times responding strongly to flooding events [27]. Collectively, these observations indicate that, since the *Synechococcus*-related ecosystem‑disruptive algal bloom (EDAB) in 2015, the trophic status of the lagoon has undergone a profound transformation [7]. However, the dynamics and ecological roles of prokaryotic communities following this initial *Synechococcus* bloom have remained largely uncharacterized.

### Shifts in prokaryotic communities in response to disruptive weather events

Microbial communities exhibit rapid responses to environmental pressures due to their short generation times, high metabolic versatility, and strong coupling to chemical and physical changes in the surrounding water. Here, we examined the prokaryotic communities of the Mar Menor over a three-year period following the deoxygenation event that occurred in October 2019, after an intense rainfall episode. Our results showed that the communities sampled at the three stations responded in a largely similar manner, with community compositions remaining comparable across most sampling dates. This contrasts with the strong spatial variability observed in the lagoon’s sediments [86], a pattern likely driven by the higher physical and chemical heterogeneity of benthic habitats, whereas the more mixed water column provides a comparatively homogeneous environment that supports more uniform microbial communities. Although it is known that nutrient-rich fresh groundwater discharge in the Mar Menor varies spatially [87] and a large area of the lagoon is currently undergoing a whiting process [88], our results show that the sampled stations adequately capture the variability of the water column across a substantial portion of the lagoon, and that the water-column communities exhibit rapid and comparable responses to short-term environmental fluctuations at all three sampled stations.

Besides spatial variability, vertical differences in microbial community composition are common in marine systems, where stratification and depth‑related gradients in light, temperature, and nutrients often structure microbial niches [89, 90]. However, the Mar Menor is a shallow lagoon, with a maximum depth of 6–7 m, and, under normal conditions, the water column remains vertically mixed [34]. As a result, the strong vertical zonation observed in deeper marine environments is not expected here. Long‑term monitoring data in the lagoon show minimal vertical gradients in temperature, salinity, and oxygen, and phytoplankton comparisons between surface and 4 m samples revealed no detectable differences (data not shown). We cannot rule out the possibility that transient stratification could, in principle, influence microbial community structure. Nevertheless, the physical characteristics of this lagoon support the use of ~ 4 m samples as representative of the entire water column in most cases.

Our study covered a wide range of environmental conditions, revealing two notable anomalous events. The first occurred in October 2019, when a ‘cold drop’ triggering heavy rainfall led to a sharp decline in oxygen and salinity levels in the lagoon. The second took place in August 2021, when oxygen levels dropped again, this time associated with high temperatures. Interestingly, while clear shifts in the relative abundance of key microbial groups were observed during the disruptive October 2019 event, no significant community‑level changes accompanied the second deoxygenation episode. During the 2019 deoxygenation event, the most striking pattern was the dominance of the cyanobacterium *Synechococcus*, which accounted for approximately 50% of the prokaryotic community and persisted dominant until February 2020, after which it virtually disappeared for several months. Notably, at that sampling time, we also detected a relatively high abundance of Actinobacteria, being the most abundant variants affiliated with Microbacteriaceae, a family rarely found in marine and, particularly coastal, environments [91, 92]. Actinobacteria, including Microbacteriaceae, exhibit metabolic versatility in carbon acquisition, allowing them to utilize diverse energy sources. In marine ecosystems, certain pelagic lineages, such as Acidimicrobiales –also present in our samples– show a preference for the deep chlorophyll maximum. This preference may reflect an opportunistic, copiotrophic lifestyle, enabling them to exploit microheterogeneity (e.g., particulate organic matter) or sporadic nutrient inputs (e.g., upwelling or deep mixing) [93]. The high abundance of Actinobacteria in the Mar Menor prior to the decline of *Synechococcus* suggests that they may have thrived on organic matter released during the decay of the *Synechococcus* peak, as observed in other systems [94].

### Seasonal patterns of alpha and beta diversity

Overall, microbial communities from October 2019 and February 2020 were significantly different from the rest of the time series, as consistently shown by the community ordination analyses (Fig. 4, Fig. S5). After this period, the communities became dominated by taxa typically found in coastal ecosystems, such as the SAR11 clade, Rhodobacterales, Pseudomonadales, and Flavobacteriales [95]. As mentioned above, during the second deoxygenation event, in August 2021, the dominant microbial taxa did not differ markedly from those present in the preceding or following months at a broad level. However, NMDS ordination revealed that these communities were compositionally distinct (Fig. S5). A more detailed examination showed shifts in the relative abundance of key ASVs. For example, an ASV affiliated with the Bacteroidetes genus *Phaeodactylibacter*, was notably abundant during this event (> 4%) compared to the rest of the dataset (average < 1%). The two deoxygenation events also resulted in contrasting alpha diversity patterns, suggesting that the ecological processes behind these events have a different nature. The first event (October 2019), associated with intense rainfall, resulted in communities exhibiting the lowest Shannon and Simpson index values of the entire study period, despite average richness (Chao1) values (Fig. S4). In contrast, the highest values for all three alpha diversity indices estimated here were observed around the second event (August 2021), linked to high temperatures. Intense rainfall and high‑temperature events may impose fundamentally different physical and biogeochemical disturbances on the water column, which could help explain, at least to some extent, the contrasting microbial responses we observed. Intense rainfall can introduce large pulses of nutrients, dissolved organic matter, and terrestrial microbes via runoff. In addition, the massive influx of freshwater can create strong salinity gradients that promote stratification of the water column. However, these storm events are often accompanied by strong winds, which can counteract stratification by inducing rapid mixing. The interplay between these opposing forces, freshwater‑driven stratification versus wind‑driven mixing, determines the extent and duration of the disturbance and ultimately shapes the response of microbial communities. These conditions may favor fast-growing taxa capable of quickly exploiting newly available resources and outcompeting slower-growing microbes [96]. In addition, strong mixing can alter nutrient stoichiometry and disrupt grazing communities (e.g., microzooplankton), reducing top-down control and thereby facilitating dominance by a single taxon, consistent with the massive *Synechococcus* proliferation and associated decrease in alpha diversity observed in October 2019. In contrast, prolonged high temperatures intensify stratification while suppressing vertical mixing. These stable, low‑mixing conditions can promote the coexistence of multiple taxa [97], particularly those with slower, resource‑conserving metabolic strategies, which aligns with the higher evenness and elevated Shannon and Simpson diversity observed in August 2021. Heat‑induced stratification may also alter grazer activity and vertical distribution [98], potentially reducing direct top‑down pressures on microbial populations. Additionally, warming affects metabolic rates, oxygen solubility, and organic matter turnover, creating conditions that may selectively favor specific functional groups. Taken together, these contrasting physical and biogeochemical regimes– coupled with the reported presence of two distinct diatom blooms during these events [27] that likely contributed different types of organic matter to the system– provide a plausible explanation for the divergent microbial outcomes observed during the two deoxygenation episodes.

Regarding seasonality, only a weak pattern was detected, in contrast with observations from other coastal Mediterranean communities [95, 99, 100] and other regions [101], where strong interannual recurrence has been reported. Although our time series is shorter and sampled less frequently than those studies, the data nevertheless show no clear annually recurring patterns in beta diversity, consistent with previous findings for eukaryotic phytoplankton communities in the lagoon [27]. The occasional, strikingly high relative abundances of cyanobacteria compared with heterotrophic bacterial taxa observed during the study period further emphasize the non-recurrent nature of microbial dynamics in the system. In agreement with these biological observations, seasonal recurrence was evident only for a subset of environmental variables.

### Shifts in *Synechococcus* abundance and dominant clades in the lagoon

Overall, the high proportions of *Synechococcus* reads in the metabarcoding analyses aligned with high *Synechococcus* cell abundances observed via flow cytometry (Figs. 2 and 3). Cell counts fluctuated by two orders of magnitude, increasing from the ‘cold drop’ in autumn 2019 (10^4^ cells mL^−1^) to December 2019 (10^6^ cells mL^−1^). Cell counts then stabilized around 10^5^ cells mL^−1^ until April 2020, before declining to lower levels (10^4^ cells mL^−1^) for the rest of the time series. In the 16S rRNA gene survey, *Synechococcus* dominated prokaryotic plankton communities in October 2019 and remained a substantial component in February 2020, before declining thereafter. However, an exception occurred in August 2021, when a peak in *Synechococcus* abundance was observed in the flow cytometry counts, but this peak was not captured in the 16S rRNA gene analyses, likely due to the lower temporal resolution of the latter. Nevertheless, the peak detected in early August declined rapidly, and by the time that the DNA sample was collected in late August, *Synechococcus* abundances measured by flow cytometry had already declined, matching the sequencing results. These observations highlight the importance of integrating multiple methodologies in long-term monitoring programs, in this case, flow cytometry and DNA sequencing. Despite the discrepancy seen in August 2021 due to differences in sampling frequency, by combining these complementary techniques, we obtained a more comprehensive and accurate understanding of microbial dynamics, improving our ability to detect short-lived population shifts and long-term ecological trends.

While partial 16S rRNA gene sequences allowed us to identify several *Synechococcus* ASVs, we characterized changes in *Synechococcus* assemblages in greater detail using the *petB* marker gene, a locus known for its higher taxonomic resolution [48, 49]. Picocyanobacteria, particularly *Synechococcus*, belong to Cluster 5 within the phylum Cyanobacteria. This cluster is divided into three main subclusters (SCs): SC 5.1 –mostly marine *Synechococcus* species, with the genus *Prochlorococcus* forming a separate branch–, SC 5.2 –a highly diverse group, including halotolerant, euryhaline, and freshwater species–, and SC 5.3 –containing both open-ocean and freshwater species– [63, 102]. Analyses by Farrant and colleagues [49] based on the *petB* marker gene provided a high‑resolution view of the ecological diversity of marine *Synechococcus* worldwide, uncovering over 20 well‑defined subclades (I-IX, XVI, XX, WPC1, Env, CRD, etc.) within subcluster 5.1, some of which are well-defined ecotypes. Our analysis revealed that the *Synechococcus* assemblages in the Mar Menor consisted of at least 11 of these subclades, including a representative of SC 5.3, with all detected *petB* variants showing sequence similarity to species isolated from marine environments (Table S4). In addition, we searched for the dominant 16S rRNA gene variants identified in our study (ASV_3, ASV_2, and ASV_28) within a recently published amplicon dataset from the Mar Menor [92], which examined the impact of submarine groundwater discharges on microbial communities in the lagoon and included samples not only from lagoon waters but also from groundwater, freshwater streams, and shore porewaters. ASV_2 and ASV_28 were detected exclusively in lagoon waters and adjacent near‑shore waters with 100% sequence similarity, whereas ASV_3 was not detected in their dataset (details not shown). These findings suggest that the increase in *Synechococcus* abundance at the beginning of the time series was driven by the response of local cyanobacterial populations, rather than by the introduction of external populations via freshwater runoff or groundwater transport following the intense rainfall event in October 2019.

The most relevant subclades represented in our dataset were subclades V, VIb, IIIa, IIe, WPC1, and subcluster 5.3 (which is not further subdivided into subclades). Farrant and colleagues [49] analyzed the global distribution patterns of these subclades to define ‘Ecologically Significant Taxonomic Units’ (ESTUs), i.e., subclades that likely occupy a common niche. For instance, subclade IIIa was identified as an ESTU primarily found in the Mediterranean Sea and the Gulf of Mexico –both known as phosphorous (P)-depleted areas– suggesting a specific adaptation to P limitation. Additionally, this ‘ecotype’ is thought to exhibit seasonality, mainly appearing during summer/autumn and/or stratified conditions. The presence of this subclade in the Mar Menor aligns with these observations, as it was mainly detected during summer and autumn over the three-year study period. The lagoon receives high nitrate loads from the catchment basin while the phosphate concentration is normally reduced; therefore, the N:P molar ratio is fairly high, possibly generating P-limited conditions for phytoplankton growth [7]. In addition, subcluster 5.3, although only sporadically detected in global datasets, has been identified as a significant component of *Synechococcus* communities in the Mediterranean and the Red Sea, where it co-occurs with subclade IIIa [49], a pattern also observed in our dataset. Another well-defined ESTU (IIB), comprising subclades IIe and IIh, has been associated with colder (14–17.5 °C) mixed waters in the Atlantic and also appears to coexist with groups IIIa and subcluster 5.3 in the Mediterranean Sea. In contrast, given the low abundance of subclades V, VI, and WPC1 in global datasets, these groups have not been delineated as ESTUs; nevertheless, they were consistently present in the Mar Menor assemblages, suggesting a notable ecological role within the lagoon.

The use of the higher-resolution phylogenetic marker *petB* allowed us to delineate and uncover the diversity masked by the 16S rRNA gene variants. In the 16S rRNA gene dataset, the same partial sequence (V4-V5 region) could match multiple subclades with 100% similarity. For instance, sequence variant ASV_2 matched subclades VIa, VIb, or VIIa, or ASV_28 matched subclades IIa, IIIa, among others (Table S3). In contrast, analysis of the *petB* gene variants revealed that the sequence likely corresponding to ASV_2 was almost exclusively associated with a single *petB* variant, *petB*_2, assigned to *Synechoccoccus* sp. PROS-7-1 from subclade VIb. Similarly, ASV_28 seemed to correspond to multiple *petB* variants distributed across subclades II and III, which exhibited mutually exclusive abundance patterns in July and October 2020 but coexisted during other sampling periods (July-November 2021; July-October 2022) (Fig. 5). Furthermore, the dominant sequence variant detected in October 2019, ASV_3, could be linked to a single *petB* variant (*petB*_1), identical to that of *Synechococcus* isolate BMK‑MC‑1, while the same ASV_3 sequence also showed 100% identity to the 16S rRNA gene of the closely related *Synechococcus* species WH7803. These results underscore the limited phylogenetic resolution of the 16S rRNA marker compared to the *petB* gene.

### Key functional traits for the growth and persistence of dominant *Synechococcus* variants

To explore the functional properties of *Synechococcus* assemblages in the lagoon, we reconstructed metagenome‑assembled genomes. Recovering cyanobacterial genomes from metagenomic data is inherently challenging due to the complexity of environmental metagenomes and the high intraspecific diversity of picocyanobacteria such as *Synechococcus*, with many closely related species often coexisting in the same environment [103]. In addition, other genomic features, such as repetitive sequences and polyploidy, further complicate assembly and binning [104]. Despite these challenges and the relatively low abundance of *Synechococcus* compared to total prokaryotic plankton at certain times, we successfully reconstructed five MAGs with >75% completeness. These MAGs seemed to be good representatives of the main genetic variants identified through the *petB* marker, as shown by the strong congruence between the temporal dynamics observed for the MAGs and the associated dominant *petB* gene variants (Fig. 5B, Fig. S7). With the exception of subclade IIIa (*petB*_3 variant), the four most abundant subclades –V, VIb, 5.3 and IIe– were captured within the reconstructed MAGs, and were taxonomically assigned to genome sequences of the *Synechococcus* cultured species WH7803, PROS-7-1, RCC307, and the environmental genome MU1617, respectively. These assignments were aligned with those determined for the *petB* variants, confirming their consistency (see Table 1). Overall, with the exception of subclades IIa, VIc, and VIII, all other subclades detected through *petB* sequences were also detected in the MAGs despite their low genome completeness (subclades IIIa, IIIb, VIa), indicating a strong congruence between both approaches.

Bacterial bloomers –prokaryotic populations that rapidly proliferate under favorable conditions– can temporarily dominate marine microbial communities, significantly influencing nutrient cycling and carbon fluxes. Several key factors contribute to this ability, including fast growth rates, resource utilization efficiency or stress tolerance and survival strategies [105]. A goal of our study was to understand the ability of the *Synechoccocus* species from the Mar Menor to proliferate in response to the impact of the intense rainfall event that occurred in autumn 2019. Besides a drop in salinity, the rainfall produced the massive runoff-driven influx of nutrients, as well as agricultural and livestock-derived contaminant products from the surrounding drainage basin into the lagoon. Notably, all *Synechococcus* species associated with the retrieved MAGs in our study possess the genetic potential to utilize urea, with the exception of strain WH7803; however, this capability is present in its close relative, species BMK-MC-1. In particular, these species contain urease genes (*ureA, ureB, ureC, ureD, ureE, ureF, ureG*) and genes encoding the urea transport system (*urtA, urtB, urtC, urtD, urtE*), which are present in most –but not all– marine, freshwater, and brackish picocyanobacteria [63]. These genes were also detected in the corresponding MAGs (data not shown), with the exception of bin.224 (related to the environmental genome MU1617), in which only genes associated with urea transport were identified. These findings support the potential ability of the *Synechococcus* assemblages detected in the lagoon to utilize organic nitrogen sources such as urea. Combined with the presence in their genomes of genes *narB* and *nirA* involved in assimilatory nitrate reduction to ammonia (ANRA) –a pathway commonly found in this cyanobacterium but less frequently found in others such as *Prochlorococcus* [106, 107]– this metabolic versatility may have provided a competitive advantage over other species, enabling *Synechococcus* to become the dominant prokaryotic taxon following the ‘cold‑drop’ event. Although marine heterotrophic bacteria also have the ability to perform assimilatory nitrate reduction, associated with the *nasA/B* gene [108], the detection of these genes in our metagenomes was negligible (data not shown).

Within *Synechococcus* assemblages, a dominant variant –recovered as MAG bin.102 and related to *Synechococcus* sp. BMK-MC-1– was prevalent in October 2019 and February 2020 (Fig. S7, Table S5). This variant carries specific genes that may have conferred an adaptative advantage for growth and persistence under the environmental conditions generated by the ‘cold drop’. A key trait was the presence of aquaporine Z (AqpZ), a water-permeable channel protein essential for maintaining cellular homeostasis [109, 110]. This protein may have protected cells from osmotic stress during the rainfall in autumn 2019. Notably, aquaporin Z is generally absent in marine *Synechococcus* species –detected only in isolate BMK-MC-1 out of the 48 marine cultured species analyzed by Cabello-Yeves and colleagues [63]– but it is widespread in freshwater and brackish picocyanobacteria. Additionally, the presence of genes associated with toxin-antitoxin (TA) systems**,** known to mediate bacterial stress responses [111, 112], were found in the dominant *Synechococcus* variant (bin.102). These systems may have helped this *Synechococcus* survive (or persist) under adverse conditions such as water deoxygenation and low irradiance through a dormancy state [113, 114], potentially explaining the rapid population increase (> 10⁶ cells mL⁻^1^) by December 2019. These TA system genes were absent in the other *Synechococcus* bins detected in this study and were generally uncommon among the 48 marine cultured species analyzed in Cabello-Yeves and colleagues [63]. In fact, some genes, like the *vapBC* toxin-antitoxin system, were exclusive to the *Synechococcus* species BMK-MC-1. Similarly, the IS5-family transposase, detected exclusively in BMK-MC-1 among marine cultured species, appears to be common in freshwater and brackish species [63]. Bloom-forming freshwater cyanobacteria frequently possess transposase‑enriched genomes [115], which contribute to genome plasticity and facilitate adaptation to fluctuating environmental conditions, including changes in nutrient availability [112, 116]. These key functional traits linked to stress tolerance of the dominant *Synechococcus* variant are more typical of brackish and freshwater cyanobacteria, which generally experience greater environmental variability than marine lineages.

Another relevant feature of MAG bin.102 was the presence of the *evrABC* genes, which encode a viologen exporter associated with herbicide resistance in cyanobacteria [85]. This system could have helped protect cells from herbicides that may have entered the Mar Menor in large quantities via runoff during the intense rainfall event [117]. In contrast, these genes were absent from the genome of MAG bin.69 and from its closest cultured relative, *Synechococcus* species PROS‑7‑1, the variant that persisted in the lagoon at low relative abundances after February 2020.

The success of bloom‑forming bacteria may also be influenced by their ability to evade grazing or resist viral infection, allowing them to persist longer in the ecosystem. Although grazing rates were not measured in this study, the dominant *Synechococcus* MAG (bin.102) was the only one that possessed an extensive and diverse repertoire of antiviral defense genes, encompassing seven distinct systems. None of these defense systems were CRISPR‑Cas systems, consistent with previous reports showing that marine *Synechococcus* typically lack CRISPR‑Cas loci [118], even though such systems are widespread in other cyanobacteria [119]. Yet, coastal *Synechococcus* species do contain abundant and diverse defense systems, which could confer them adaptability to the dynamic coastal environment [118]. High host density increases the likelihood of virus-cell encounter and subsequent rapid multiplication of viruses. The genomes of bloom-forming species are thus expected to be enriched in antiviral genes to counter the likely attack by phages [120]. Despite these genes allow certain bacterial species to persist longer, phages eventually overcome the defenses ─e.g., phages evade the restriction-modification system by accidental methylation of viral DNA [121]─ and play a crucial role in terminating bacterial blooms through lysis, as discussed below. The unusually broad defensive arsenal of bin.102 suggests that this *Synechococcus* variant may have been under strong viral predation pressure and has acquired mechanisms to withstand phage attack.

Altogether, these results indicate that the presence of genes related to osmotic stress tolerance, toxin–antitoxin systems, herbicide resistance, and an extensive and diverse repertoire of antiviral defense systems, may have conferred *Synechococcus* MAG bin.102 a competitive advantage over other variants in the aftermath of the autumn 2019 intense rainfall event, enabling its proliferation and persistence in the lagoon between October 2019 and February 2020.

### Phage control over *Synechococcus* populations

As virus-host interactions can strongly influence the dynamics of marine bacteria, including cyanobacteria [33], we analyzed prokaryotic viruses in the same picoplankton metagenomes used for our prokaryotic analyses, with a particular focus on cyanophages (viruses infecting cyanobacteria). Although some viral sequences could derive from extracellular, morphologically complete particles (“virions”) retained on the filters because of their large size (e.g., jumbo phages), most viruses in this fraction are expected to originate from infections occurring within host cells. We found that cyanophages infecting *Synechococcus* were an important component of the Mar Menor viral assemblages, displaying relative abundances that largely covaried with those of their host over time (Fig. 5). This pattern is consistent with the rapid response of phages to host availability, supporting their role as immediate top‑down regulators of *Synechococcus* populations through lytic infection. The decline in cyanophage relative abundance from October 2019 to October 2020, suggests that viruses played a significant role in terminating the host abundance peak detected following the 2019 ‘cold drop’ event. It is possible that the accumulation of free viral particles following the collapse of *Synechococcus* populations, and their unintended retention in the cellular fraction during filtration (potentially due to filter clogging), contributed to the observed decline in *Synechococcus*– and subsequently cyanophages– from December 2020 to April 2021. Such conditions can promote the emergence of a distinct dominant species, as previously observed in the Red Sea [122], but this did not occur in our system. Despite *Synechococcus* reaching its highest abundances between October 2019 and February 2020, the largest peak in cyanophage relative abundance occurred later, in July 2022, in the absence of host abundance peaks. This delayed maximum could reflect a combination of accumulated extracellular virions from earlier infection events and a higher proportion of host variants present at that time that were susceptible to viral infection.

During blooms of marine microbes, community‑level diversity commonly declines as one or a few particularly successful species dominate [123]. Consistent with this pattern, the cyanophage community in October 2019, coinciding with the early stage of the *Synechococcus* proliferation, exhibited the lowest richness among all samples associated with viral abundance peaks (i.e., those with relative abundance above 0.25%). It was also the least even community overall. This pattern suggests the dominance of specific viral variants, likely promoted by the rapid increase of particular host lineages at that time, which would have imposed strong selective pressures on the viral community [124]. The subsequent increase in cyanophage richness and evenness from February to July 2020, coupled with viral relative abundances toward the decline of the *Synechococcus* peak within the same period, supports the hypothesis that the initially dominant *Synechococcus* variant was gradually replaced by other lineages over this period (in agreement with the host marker gene dynamics shown in Fig. 5). It is plausible that viral pressure contributed to the decline of the dominant host lineage, paving the way for a more diverse community of susceptible host variants and, consequently, a richer phage assemblage. Altogether, these results highlight cyanophages as key drivers shaping the composition and succession of *Synechococcus* communities in the lagoon.

## Concluding remarks

This study represents the first time series analysis of prokaryotic communities in the waters of the Mar Menor and provides insights into how these communities respond to environmental disturbances in the lagoon. Our findings reveal that water‑column prokaryotic communities respond rapidly and in a largely uniform manner to short‑term environmental fluctuations. The analysis of communities during two deoxygenation events, October 2019 and August 2021, triggered by distinct extreme weather conditions (intense rainfall and high temperatures, respectively) revealed contrasting microbial diversity responses, underscoring the complexity of the ecosystem's resilience. While the 2019 event led to reduced microbial diversity and the dominance of *Synechococcus* variants, the 2021 event resulted in increased diversity, dominated by members of the Alpha‑ and Gammaproteobacteria, suggesting different ecological drivers and adaptive responses. The weak seasonal trends in community alpha and beta diversity further point to an altered ecosystem shaped by recurring anthropogenic and climatic pressures. The dominance of *Synechococcus* following the ‘cold drop’ of autumn 2019 highlights the capacity of this cyanobacterium to exploit nutrient influxes and cope with sudden environmental change. Genetic and genomic analyses suggest that the dominance of a specific *Synechococcus* variant in the lagoon -closely related to the isolated species *Synechococcus* BMK‑MC‑1- was likely facilitated by a suite of functional traits, including mechanisms for osmotic stress tolerance, toxin–antitoxin systems, herbicide resistance, and diverse antiviral defense mechanisms. Together, these features may have conferred a competitive advantage in the aftermath of the disturbance, enabling this variant to proliferate while other microbial taxa were less able to thrive in the altered environmental conditions. Moreover, the analysis of prokaryotic viruses suggests that cyanophages play a major role in shaping the composition and succession of *Synechococcus* assemblages in the lagoon.

On the other hand, this study highlights the importance of integrating multiple methodological approaches, including DNA sequencing, flow cytometry, and the analysis of different planktonic compartments (i.e. viruses and prokaryotes), to capture microbial community dynamics across temporal scales. Expanding these efforts to include grazers would further improve our understanding of the top‑down controls acting on microbial populations. The successful reconstruction of *Synechococcus* MAGs provides valuable genomic insights into the adaptive strategies of bloom‑forming bacteria, with broader implications for understanding microbial resilience in coastal ecosystems. Identifying the functional traits that enable microbial populations to thrive under stress will be essential for predicting ecosystem responses to future disturbances. Given the ongoing environmental pressures affecting the Mar Menor, continued long‑term monitoring that integrates microbiome analyses with broader ecological assessments will be crucial for effective management and conservation of this vulnerable ecosystem.

## Supplementary Information

Supplementary file 1.

Supplementary file 2.

## Data Availability

Amplicon sequence data of the 16S rRNA gene have been deposited in the European Nucleotide Archive under the project accession number PRJEB88874. Sequences of the *Synechococcus* MAGs, *petB* gene variants, and cyanophages, together with their corresponding abundance tables, are available at: https://github.com/AnaMariaCabello/Mar_Menor_picoplankton_dynamics, a repository that also includes the abundance table of the 16S rRNA gene amplicon sequence variants (ASVs).
