# Supplementary material for: Environmental disturbances and cyanobacterial traits shape prokaryotic dynamics in a eutrophic Mediterranean coastal lagoon
Source: Environ Microbiome. 2026 Apr 29;21:89. doi: 10.1186/s40793-026-00893-9 (PMC13366656; doi:10.1186/s40793-026-00893-9)
Supplement: Supplementary file 2 [file 40793_2026_893_MOESM2_ESM.pdf]

Supplementary Materials

Cabello *et al.* Environmental Microbiome

Environmental Disturbances and Cyanobacterial Traits Shape Prokaryotic Dynamics in a Eutrophic Mediterranean Coastal Lagoon

Supplementary Figures:

Fig. S1. Monthly data of abiotic variables

Fig. S2. Monthly data of nutrient concentrations

Fig. S3. Monthly data of biotic variables

Fig. S4. Alpha diversity values

Fig. S5. NMDS plots

Fig. S6. Correlations between *Synechococcus* variants and environmental variables

Fig. S7. Temporal dynamics of *Synechococcus* MAGs

Fig. S8. Heatmap of *Synechococcus*-infecting vOTUs

Supplementary Tables*:

Table S1. Lomb-Scargle periodograms for environmental variables

Table S2. dbRDA permutation test results

Table S3. BLAST similarity of top *Synechococcus* ASVs

Table S4. BLAST similarity of *Synechococcus petB* metagenomic sequences

Table S5. *Synechococcus* MAG bin quality and taxonomic affiliation

Table S6. Genes unique to *Synechococcus* sp. BMK-MC-1

Table S7. Antiviral defense genes in *Synechococcus* MAGs

Table S8. Host predictions for *Synechococcus*-associated viruses

*As separated downloadable file

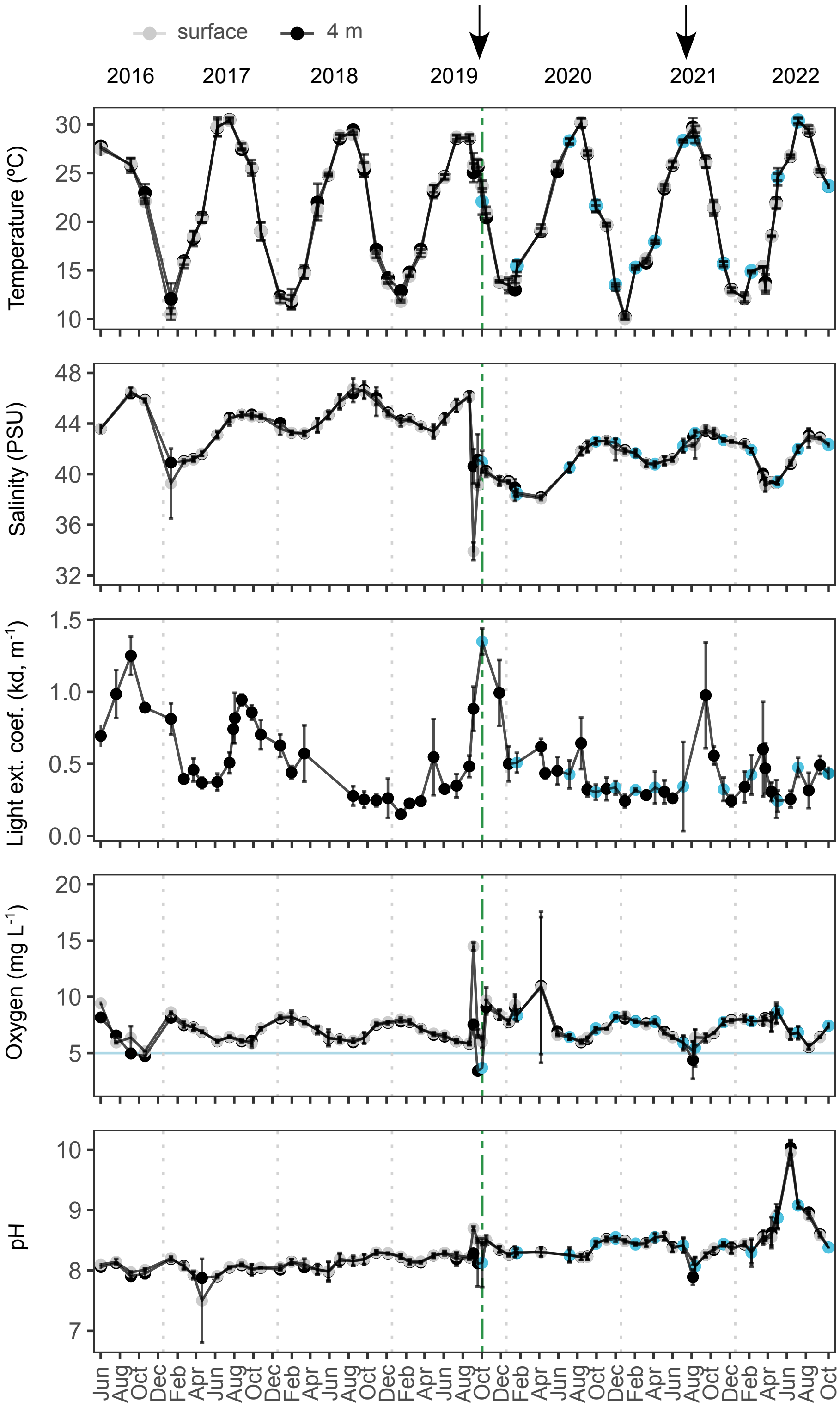

Fig. S1. Monthly data of abiotic variables measured in the lagoon from June 2016 to October 2022. Data represent averages from stations A, B, and C at the surface (grey dots) and at ~4 m depth (black dots); error bars show standard deviation. The green dashed line marks the start of DNA sample collection (October 2019), and blue dots indicate the specific dates on which these samples were obtained. In the oxygen panel, the horizontal blue line indicates the hypoxia threshold. Months are shown on the x-axis and years are indicated above the top panel. Arrows above the top panel mark the timing of the two deoxygenation events.

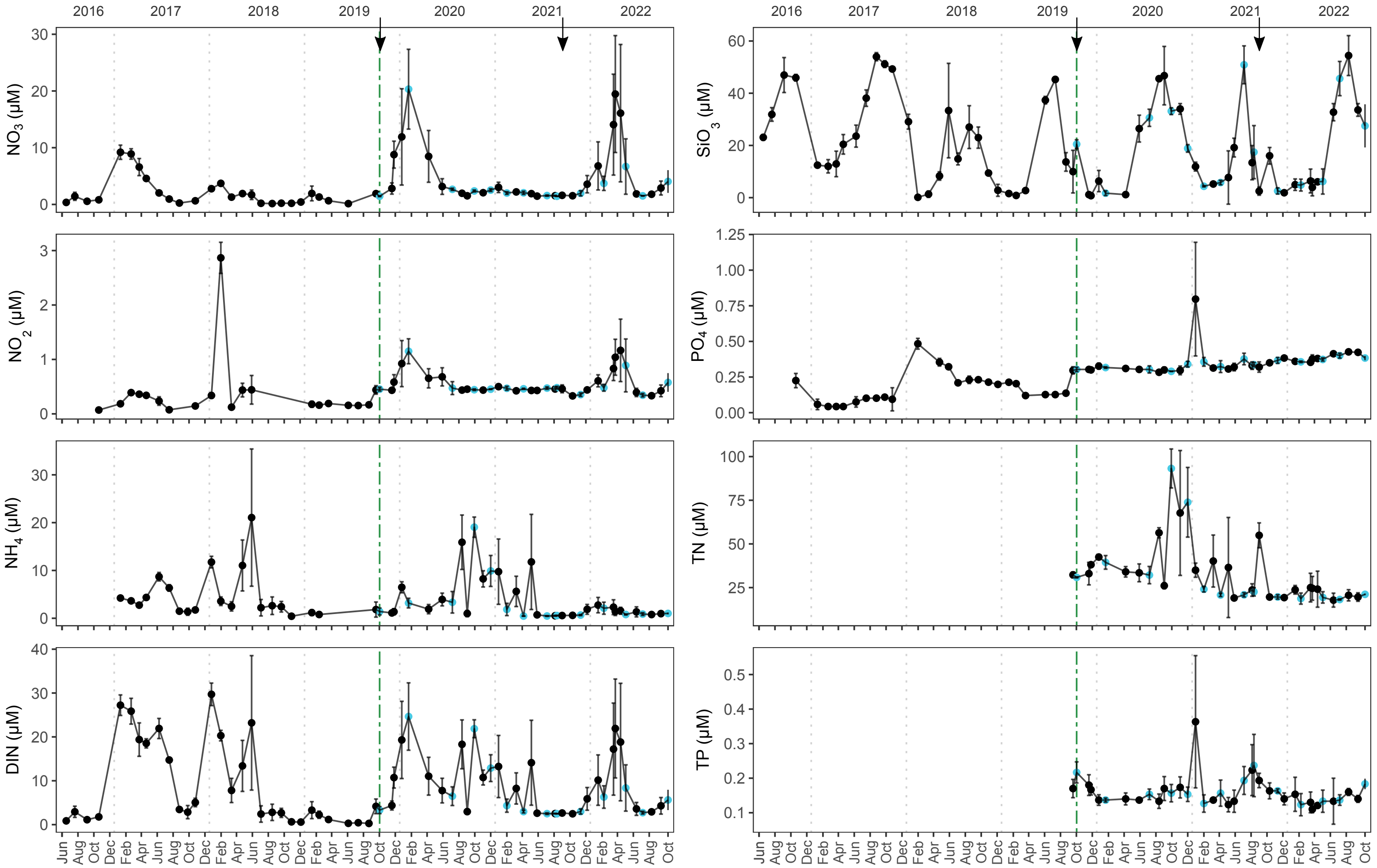

Fig. S2. Monthly data of nutrient concentrations measured in the lagoon from June 2016 to October 2022. Data represent averages from stations A, B, and C at ~4 m depth (black dots); error bars show standard deviation. The green dashed line marks the start of DNA sample collection (October 2019), and blue dots indicate the specific dates on which these samples were obtained. Months are shown on the x-axis and years are indicated above the top panel. Arrows above the top panel mark the timing of the two deoxygenation events. Part of the nutrient (up to 2021) were previously published in Mercado *et al.* 2021 [7].

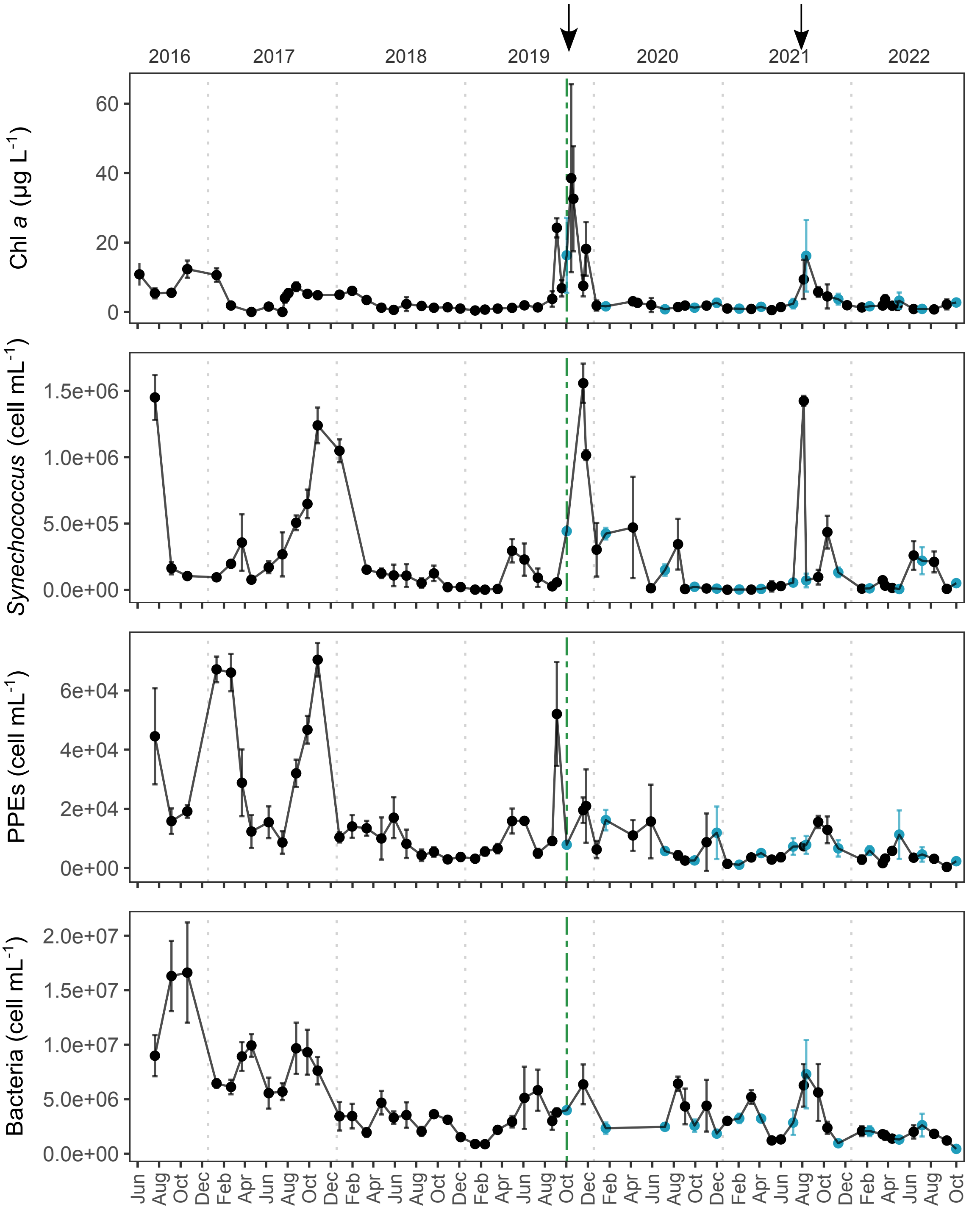

Fig. S3. Monthly data of biotic variables measured in the lagoon from June 2016 to October 2022. Data represent averages from stations A, B, and C at ~4 m depth (black dots); error bars show standard deviation. The green dashed line marks the start of DNA sample collection (October 2019), and blue dots indicate the specific dates on which these samples were obtained. Months are shown on the x-axis and years are indicated above the top panel. Arrows above the top panel mark the timing of the two deoxygenation events. Part of the chlorophyll *a* concentration and *Synechococcus* abundance data (up to 2021) were previously published in Mercado *et al*. 2021 [7] and Ouaissa *et al.* 2023 [27].

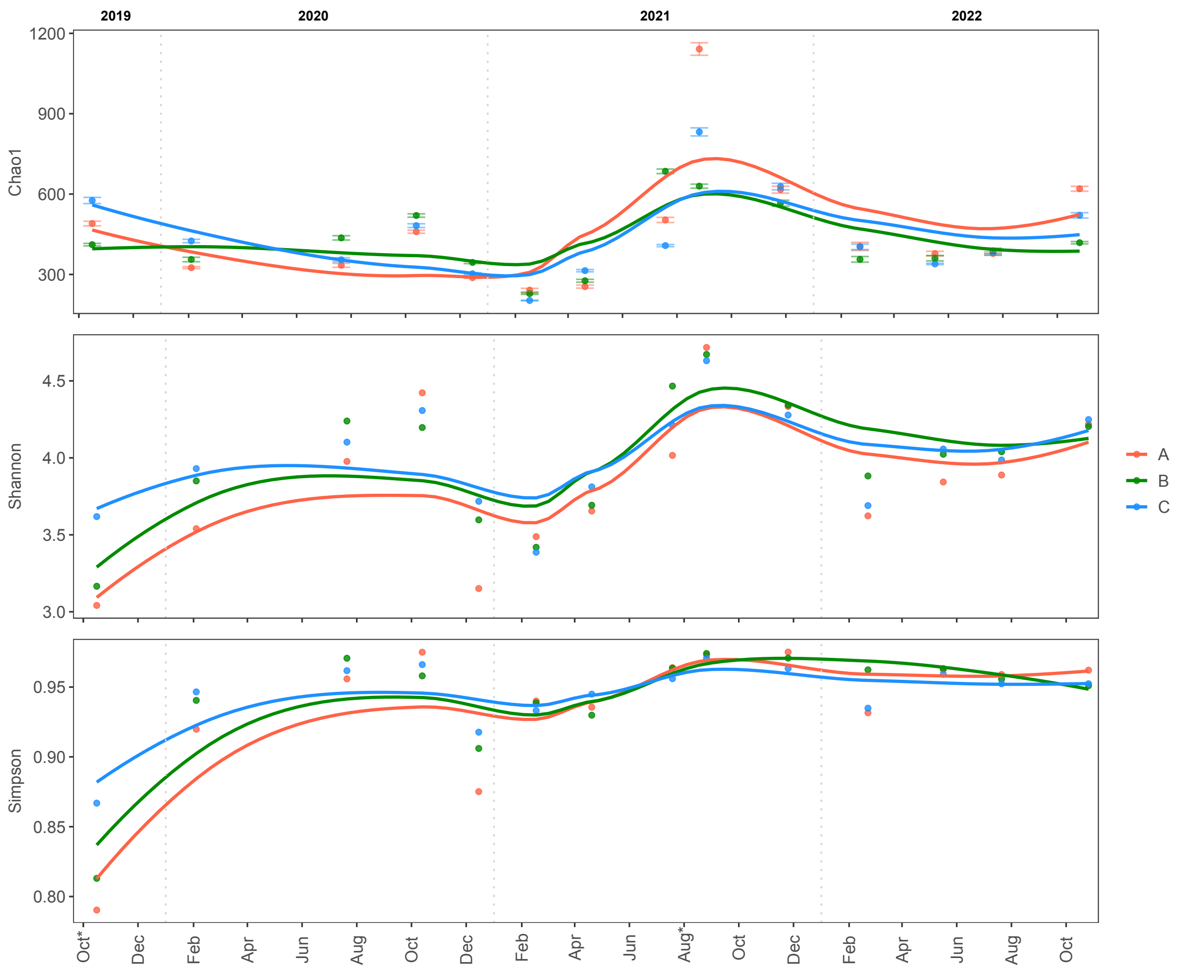

Fig. S4. Alpha diversity metrics (Chao1 richness, Shannon, and Simpson indices) color-coded by station across the sampling period. The x-axis shows the month of sampling, and the year is indicated above the top panel. Asterisks denote the dates of the two deoxygenation events.

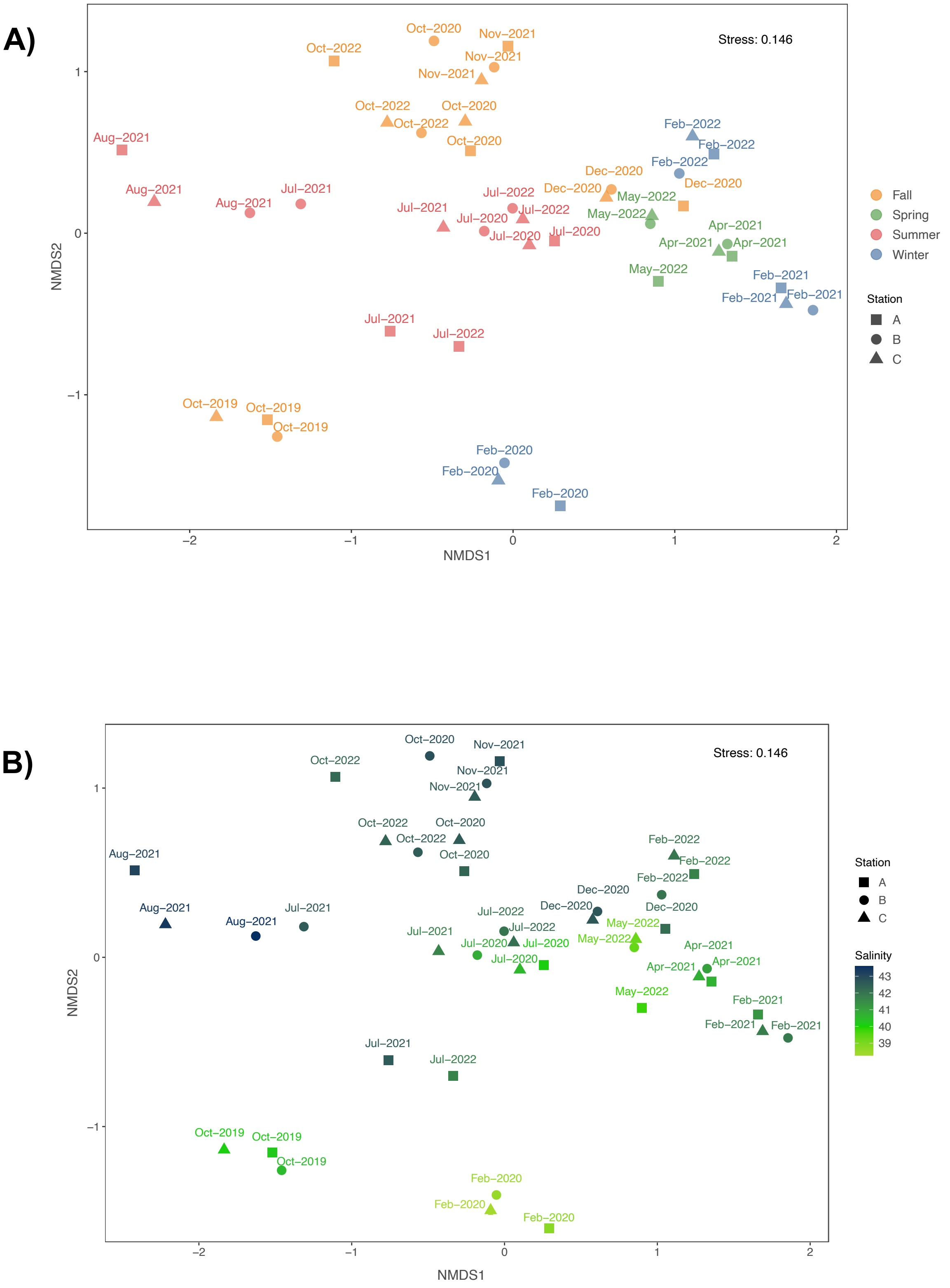

Fig. S5. Non-metric Multidimensional Scaling (NMDS) plots based on Bray–Curtis dissimilarity distances of prokaryotic communities, showing the ordination of samples collected between October 2019 and October 2022 at stations A, B, and C (represented by different symbols). Plots are color-coded by season (A) or by salinity values (B).

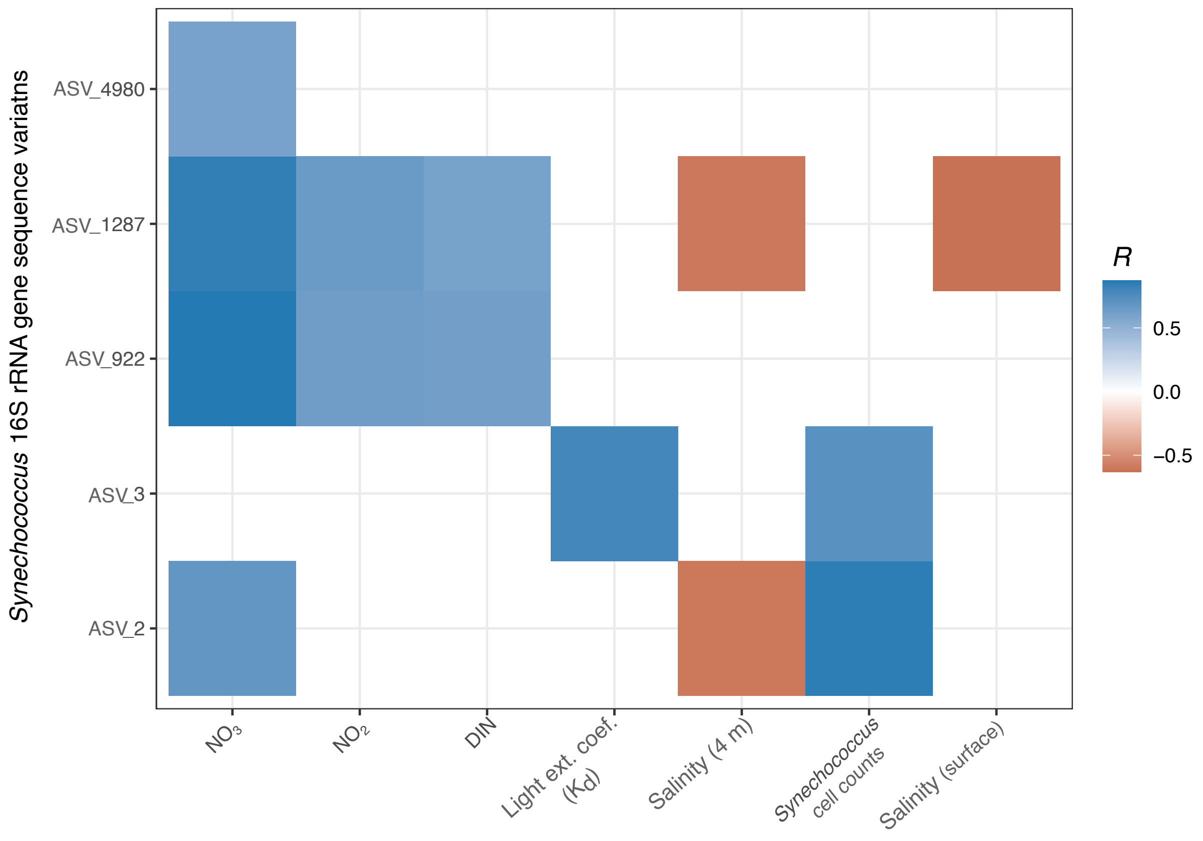

Fig S6. Pearson correlation coefficients between the relative abundance of *Synechococcus* 16S rRNA gene variants and environmental variables. The plot only displays statistically significant correlations ($p<0.05$, $n = 42$). DIN: dissolved inorganic nitrogen.

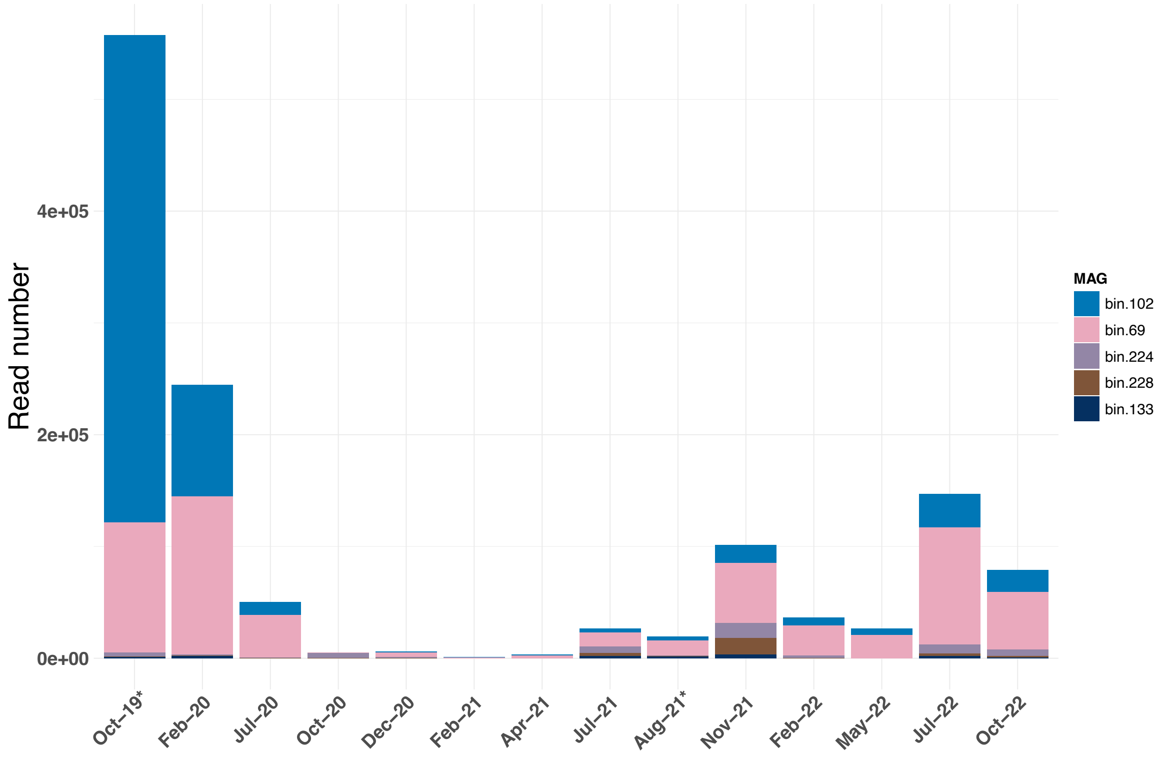

Fig. S7. Temporal dynamics of *Synechococcus* MAG relative abundances. Read abundances (normalized by contig length) mapped to each MAG are shown. MAGs are color coded according to their GTDB assigned *Synechococcus* subclades. Asterisks indicate the dates of the two deoxygenation events.

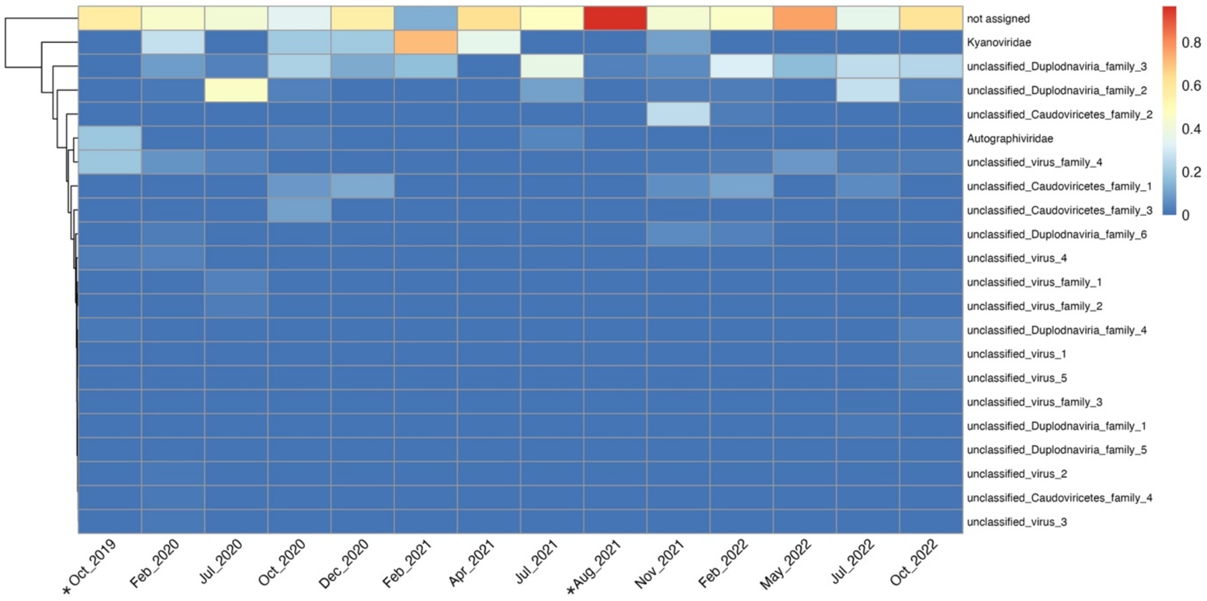

Fig. S8. Heatmap of the relative abundances of *Synechococcus*-infecting vOTUs aggregated by viral taxon across the Mar Menor time series, hierarchically clustered by abundance patterns. Relative abundances were calculated per sample as the summed abundance of vOTUs within each taxon divided by the total abundance of all vOTUs predicted to infect *Synechococcus*. Representative contigs of vOTUs shorter than 10 kb, which could not be assigned to viral taxa, were grouped in the 'not assigned' category.
